# Ferroptosis regulation through Nrf2 and implications for neurodegenerative diseases

**DOI:** 10.1007/s00204-023-03660-8

**Published:** 2024-01-24

**Authors:** Yao Xiang, Xiaohua Song, Dingxin Long

**Affiliations:** 1 School of Public Health, Hengyang Medical School, University of South China, Hengyang, 421001 People’s Republic of China; 2 Hunan Province Key Laboratory of Typical Environmental Pollution and Health Hazards, Hengyang Medical School, University of South China, Hengyang, 421001 People’s Republic of China

**Keywords:** Neurodegenerative diseases, Ferroptosis, Oxidative stress (OS), Regulation, Nuclear factor E2-related factor 2 (Nrf2)

## Abstract

This article provides an overview of the background knowledge of ferroptosis in the nervous system, as well as the key role of nuclear factor E2-related factor 2 (Nrf2) in regulating ferroptosis. The article takes Alzheimer's disease (AD), Parkinson's disease (PD), Huntington's disease (HD), and amyotrophic lateral sclerosis (ALS) as the starting point to explore the close association between Nrf2 and ferroptosis, which is of clear and significant importance for understanding the mechanism of neurodegenerative diseases (NDs) based on oxidative stress (OS). Accumulating evidence links ferroptosis to the pathogenesis of NDs. As the disease progresses, damage to the antioxidant system, excessive OS, and altered Nrf2 expression levels, especially the inhibition of ferroptosis by lipid peroxidation inhibitors and adaptive enhancement of Nrf2 signaling, demonstrate the potential clinical significance of Nrf2 in detecting and identifying ferroptosis, as well as targeted therapy for neuronal loss and mitochondrial dysfunction. These findings provide new insights and possibilities for the treatment and prevention of NDs.

## Introduction

**Fig. 1:**
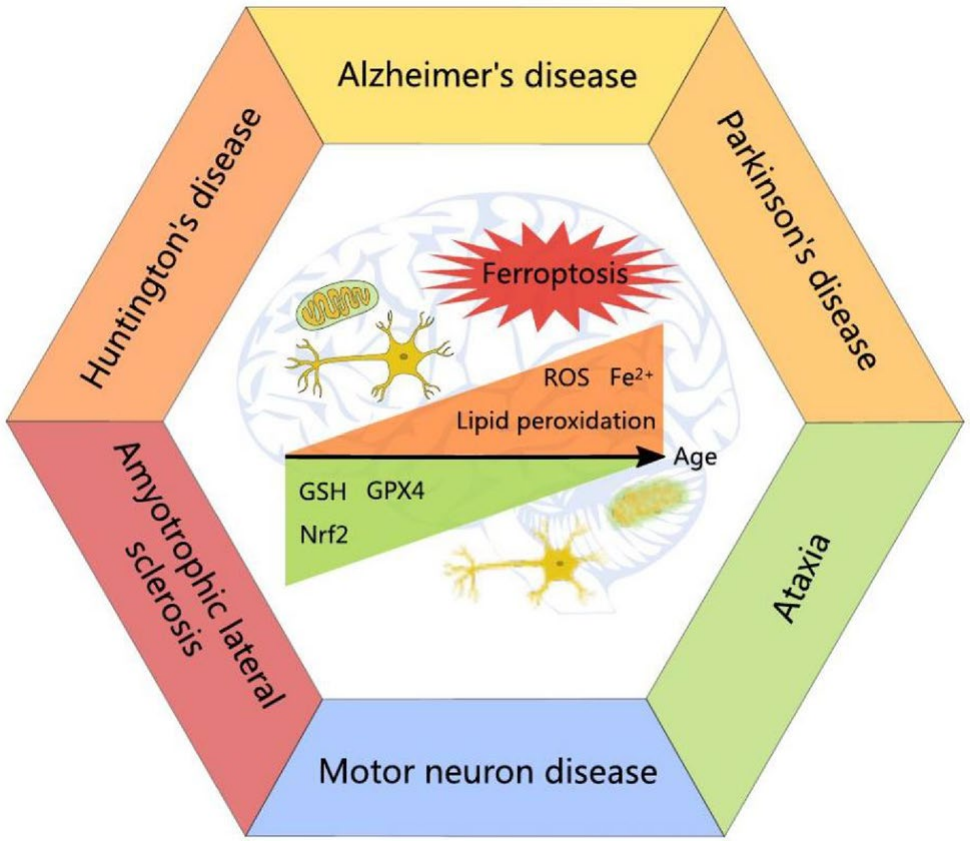
Characterization of ferroptosis in neurodegenerative diseases (NDs) pathological conditions. There exists a significant correlation between ferroptosis and NDs. Ferroptosis contributes to the advancement of NDs, whereas the expression of Nrf2 and its transcriptionally regulated peptides (GSH, GPX4) decreases with aging. In contrast to the impairment of the antioxidant system, heightened levels of reactive oxygen species (ROS) and increased lipid peroxidation, combined with brain iron deposition, induce ferroptosis. Ferroptosis results in alterations in mitochondrial morphology, neuronal damage and eventual cell death. Drawing by Inkscape

Alzheimer's disease, Parkinson's disease, Huntington's disease, and amyotrophic lateral sclerosis are typical neurodegenerative diseases (NDs) (Maragakis and Rothstein 2006) that can be identified by the gradually implemented death of specifically vulnerable neurons (Abdalkader et al. 2018). Today, the global prevalence of Alzheimer's disease (AD) stands at over 50 million individuals, with projections indicating that this number will surpass 100 million by the mid-century (Jannat et al. 2019). Elderly people are more likely to develop common NDs like Alzheimer's ( AD) and Parkinson's disease, or PD, and their risk increases with age (Hou et al. 2019). Among the many risk factors for neurodegeneration, the impact of aging cannot be ignored (Wyss-Coray 2016; Dugger and Dickson 2017). With the aging of the world's population, age-related NDs have become one of the biggest problems to be solved urgently in modern society (Angelova et al. 2021). To improve patient quality of life, increase life expectancy, and lessen the burden on society, it is crucial to understand the pathogenesis of NDs and identify effective therapeutic targets. At the same time, a considerable number of studies have shown an association between the onset and progression of NDs and ferroptosis (Gallucci et al. 2008; Wu et al. 2021; Zhang et al. 2023) (Fig. 1).

As originally suggested by Dixon's groups in the year 2012 ferroptosis is now recognized as an iron-dependent type of lipid peroxidation-induced programmed cell death (Dixon et al. 2012). The morphological changes and biochemical processes that differentiate ferroptosis from other forms of programmed cell death are indicative of the unique characteristics of this process (Lee et al. 2020). Morphologically, it is characterized by ferroptosis-like nanopores in cell membrane, mitochondrial shrinkage, mitochondrial exterior membrane rupture and elevated membrane of the mitochondria density (Dixon et al. 2012; Stockwell 2022), mitochondrial ridge reduction or loss, and no significant changes in nucleus (Demuynck et al. 2021; Dong et al. 2022). In biochemical terms, iron excess, lipid peroxidation, increased ROS, and impaired antioxidant enzyme activity are the hallmarks of ferroptosis (Jiang et al. 2021). Lipid peroxidation is the procedure that happens when specific phospholipids in cell and organelle membranes attach to reactive chemicals such as reactive oxygen species, or ROS for short, and reactive nitrogen species (RNS) [mainly from the combination of polyunsaturated fatty acids with phospholipids, especially arachidonic acid and epinephric acid (AA), which can bind to phosphatidylethanolamines (PE)] to generate lipid peroxides (LOOH) (Dodson et al. 2019a). The increase of free iron leads to Fenton reaction (Fe^2+^ + H_2_O_2_ → Fe^3+^  + ·OH + OH^−^), resulting in ROS accumulation and non-enzymatic lipid peroxidation, which leads to the imbalance of antioxidant system and membrane integrity defect, and finally leads to ferroptosis (Forcina and Dixon 2019) (Fig. 1).

Among them, the main antioxidant systems currently involved include: the Xc^−^–glutathione–glutathione peroxidase 4 axis (Xc^−^–GSH–GPX4) (Yang et al. 2014), coenzyme Q_10_–ferroptosis suppressor protein1 (CoQ_10_–FSP1) (Doll et al. 2019), dihydroorotate dehydrogenase–ubiquinol (DOHDH–CoQ_10_H_2_) (Mao et al. 2021) and GTP cyclohydrolase1–tetrahydrobiopterin (GCH1–BH_4_) axis (Kraft et al. 2020). Significantly, Nrf2 regulates the transcription of the anti-ferroptosis system, encompassing crucial signaling molecules involved in these pathways. Thus, it is thought that Nrf2 is a crucial regulator of ferroptosis and peroxidation of lipids (Dodson et al. 2019a). In the following article, we emphasize how ferroptosis and the control of Nrf2 signaling are related, with its associated iron metabolism and lipid peroxidation. Based on the research on the mechanism of ferroptosis in NDs, discuss the feasible strategies and related biomarkers of Nrf2 regulatory pathway in the diagnosis and treatment of ferroptosis-driven neurodegeneration.

## Iron metabolism

Iron is the most prevalent transition metal on the earth (Coby et al. 2011) and the most abundant essential metal in the organism (Wang et al. 2022b), which defines an important role in a variety of physiological processes and even in the origin of life. As a transition metal, iron has efficient electron transfer properties, thus participating in the synthesis of heme and assisting in catalyzing various biochemical reactions (Crielaard et al. 2017). According to related research, metabolic reactions involving iron or iron–sulfur clusters may have played a key role in the development of life (Bonfio et al. 2017; Varma et al. 2018). In the body, nearly half of the iron constitutes the active center of hemoglobin, thus participating in oxygen transport (Andreini et al. 2018). About 20% of the total oxygen is consumed by the relatively small brain separated by the blood–brain barrier (BBB) (Angelova et al. 2021). Furthermore, the development of synapses, myelination, and the synthesis and turnover of neurotransmitters all depend on iron-dependent protein and enzyme activity in the nervous system (Carpenter et al. 2016). Iron homeostasis profoundly affects brain development and health (Masaldan et al. 2019). The effect of iron deficiency on brain functions such as intelligence and cognition is particularly significant in the early stages of development, even when the condition is corrected (Beard and Connor 2003).

At the same time, the presence of the BBB, which separates the brain from the peripheral iron homeostatic storage, in turn influences the import of iron into the brain (Masaldan et al. 2019). Brain capillary endothelial cells (BCECs) are a major component of the BBB (Masaldan et al. 2019) (Fig. 3a), and the transferrin receptor 1 (TfR1) expressed on them is responsible for the transfer of iron into the brain (Pardridge et al. 1987; Connor et al. 2011). In vivo, free iron usually has two states: ferric (Fe^3+^) and ferrous (Fe^2+^) irons (Pan et al. 2021). Fe^3+^ forms a complex (holo-Tf) with transferrin (Tf) after entering the blood circulation, and binds to TfR1 on the surface of brain microvascular endothelial cells (BMECs), and then the Fe^3+^-containing Tf–TfR1 complex enters the BME through clathrin-mediated endocytosis (Pardridge et al. 1987; Taylor et al. 1991; McCarthy and Kosman 2012) (Table 1).

**Table 1:** Signaling molecules associated with ferroptosis and regulated by Nrf2

Pathway	Signaling molecule	Description	Function	Regulate by Nrf2	References
Iron metabolism pathway	FTH1	Ferritin heavy chain 1	Subunit of ferritin; oxidize Fe^2+^ to Fe^3+^	↑	Chen et al. (2020)
	FTL	Ferritin light chain	Subunit of ferritin; promotes the entry of free iron into the ferritin mineral core	↑	
	Tf	Transferrin	Binds to Fe^3+^ in the circulation to form a holo-Tf complex	Unknown	Pardridge et al. (1987) and Taylor et al. (1991)
	TfR1	Transferrin receptor 1	Imports Fe^3+^ into cells by binding to the holo-Tf complex	Unknown	
	STEAP3	Six transmembrane epithelial antigen-prostate 3	Reduces Fe^3+^ to Fe^2+^ in an acidic environment	Unknown	Ohgami et al. (2005)
	DCYTB	Duodenal cytochrome b	Reduces Fe^3+^ to Fe^2+^ in an acidic environment	Unknown	Tulpule et al. (2009)
	DMT1	Divalent metal transporter 1	Transports Fe^2+^ from nuclear endosomes to the cytoplasm	Unknown	De Domenico et al. (2008)
	FPN1	Ferroportin 1, solute carrier family 40 member 1 (SLC40A1)	Export excess iron out of cells	↑	Drakesmith et al. (2015)
	Cp	Copper cyanine	Oxidizes Fe^2+^ to Fe^3+^	Unknown	McCarthy and Kosman (2013) and Burkhart et al. (2016)
	Heph	Hephaestin	Oxidizes Fe^2+^ to Fe^3+^	Unknown	
	HO1	Heme oxygenase 1, HMOX1	Catalyzes the heme's breakdown to yield equimolar levels of Fe^2+^, biliverdin and CO	↑	Loboda et al. (2016)
*Antioxidant system*					
GSH–GPX4 axis	SLC3A2	Solute carrier family 3 member 2	Maintains the structural stability of System Xc^−^; a light-chain xCT protein of System Xc^−^	Unknown	Koppula et al. (2018)
	SLC7A11	Solute carrier family 7 member 11	Import cystine in the cell; functional component of System Xc^−^	↑	Sato et al. (1999) and Liu et al. (2021)
	TXNRD1	Selenium-containing thioredoxin reductase	Reduces ingested cystine to cysteine	↑	Ursini and Maiorino (2020)
	GCL	Glutamate-cysteine ligase	Key enzyme involved in GSH synthesis	↑	Seibt et al. (2019) and Yan et al. (2022)
	GSS	Glutathione synthetase	Key enzyme involved in GSH synthesis	↑	
	GPX4	Glutathione peroxidase 4	Converts lipid hydroperoxides to nontoxic lipid alcohols via GSH depletion	↑	Yang et al. (2014)
	ME1	Malate 1	Reduces NADP^+^ to NADPH via the pentose phosphate pathway	Unknown	Fang et al. (1970)
	GSR	Glutathione reductase	Catalyzes GSSG reduction to GSH by using NADPH as a reducing cofactor	Unknown	Wu et al. (2011) and Ammal Kaidery et al. (2019)
FSP1–ubiquinone (CoQ_10_) axis	FSP1	AIFM2, AMID, PRG3	NAD(P)H-dependent ubiquinone oxidoreductase and vitamin K reductase at the plasma membrane	↑	Bersuker et al. (2019) and Mishima et al. (2022)
GCH1–BH_4_–phospholipid axis	GCH1	GTP cyclohydrolase 1	Key enzyme in the endogenous synthesis of tetrahydrodiphosphate (BH_4_)	↑	Kraft et al. (2020)
Other	NQO1	NAD(P)H: quinone oxidoreductase 1	NAD(P)H-dependent dehydrogenase (quinone) reductase; superoxide reductase	↑	Siegel et al. (2004) and Ross and Siegel (2017)
	DHODH	Dihydroorotate dehydrogenase	Mediate the reduction of ubiquinone to keep ubiquinone levels in mitochondrial membranes	↑	Mao et al. (2021)
Lipid peroxidation	ACSL4	Acyl-CoA synthetase long-chain family member 4	Activates PUFAs to generate long-chain fatty acyl-CoA (PUFA-CoA)	Unknown	Yuan et al. (2016) and Doll et al. (2017)
	LPCAT3	Lysophosphatidylcholine acyltransferase 3	Inserts the acyl group into the hemolytic phospholipid (PUFA-PE)	Unknown	
	LOX	Lipoxygenases	Oxidizes PUFA on the membrane (PUFA-PE)	Unknown	Wenzel et al. (2017) and Chu et al. (2019)

The transport mechanism of iron across BCEC is still inconclusive, but there are two possible scenarios (Masaldan et al. 2019). Classical models hold that Fe^3+^ released from Tf in an acidic environment created by endosomal proton pumps is reduced to Fe^2+^ by six transmembrane epithelial antigen-prostate 3 (STEAP3) (Ohgami et al. 2005) or duodenal cytochrome b (DCYTB) (Tulpule et al. 2009), then transported to the cytoplasm by divalent metal transporter 1 (DMT1) (De Domenico et al. 2008), while apotransferrin (apo-Tf; i.e., iron-free transferrin) and TfR1 are recovered by the cell membrane for recycling (Zheng et al. 2018; Chen et al. 2019). In this case, one of the multiple destinations of Fe^2+^ in BMECs is to be transported out of the cell via the secretion of ferroportin 1 (FPN1) to the brain, where it is subsequently oxidized to Fe^3+^ by copper cyanine (Cp) or heparin (McCarthy and Kosman 2013; Burkhart et al. 2016) (Fig. 3d). The classical model could not explain the finding of DMT1 deficiency on BMECs, so another model of transcytosis was proposed (Moos et al. 2006; Burkhart et al. 2016). In this model, holo-TF, a complex of Fe^3+^ with Tf, is directly transported through vesicles to abluminal site, thus released into the brain (Raub and Newton 1991; Moos et al. 2007) (Fig. 3b). The fact that in vitro nurtured bovine BCECs cycle Tf-TfR1 complexes may not result in intraendothelial breakdown in transcytosis is one argument in favor of the transcytosis paradigm (Raub and Newton 1991; Descamps et al. 1996). Yet, no additional in vivo studies support the belief that circulating Tf translocates through BCECs and enters the brain (Crowe and Morgan 1992; Strahan et al. 1992; Moos et al. 2006). In the classical model, Fe^2+^ is involved in a variety of biochemical processes within the BCEC, including efflux. There are two storage forms of iron in the cell: stable ferritin and active unbound iron, namely the unstable iron pool (LIP) (Ru et al. 2023). Excess reactive Fe^2+^ as LIP can cause ROS accumulation through Fenton reaction (Yan et al. 2021b). Finally, neurons, microglia, and astrocytes can utilize the same iron metabolism-related proteins to absorb iron across the BBB (Simpson et al. 2015; McCarthy and Kosman 2015; Bu et al. 2019; Yan and Zhang 2019) (Fig. 3d).

Astrocytes are primarily responsible for releasing iron into neurons while attenuating iron toxicity (Abbott et al. 2006; Burkhart et al. 2016). Cp is required for iron export from astrocytes (Klomp et al. 1996; Jeong and David 2006), whereas the processes associated with their iron import are unknown, and the expression level of TfR1 in these cell varies in different studies (Pelizzoni et al. 2013; Wong et al. 2014a; Zarruk et al. 2015; Belaidi and Bush 2016). DMT1 may have a facilitative effect on the transfer of iron into astrocytes (Moos and Morgan 2003; Huang et al. 2004, 2006; Lane et al. 2010; Pelizzoni et al. 2013). The concentration of iron in glial cells is higher than that in neurons, and the maintenance of iron homeostasis in the central nervous system mainly depends on astrocytes (Dusek et al. 2022), which accumulate iron and iron-containing compounds and store them efficiently in ferritin export iron (Hohnholt and Dringen 2013; Codazzi et al. 2015; Morris et al. 2018). Studies have also shown that astrocytes in the mouse brain can activate Nrf2 to resist cytotoxicity caused by iron overload (Cui et al. 2016). The neuroprotective effect mediated by Nrf2 has also been observed in various experimental models of neurodegeneration (Cuadrado et al. 2018; Yu et al. 2019) In addition, solute carrier family 39 member 14 (SLC39A14) can also transport Fe^2+^ intracellularly (Yu et al. 2020), and its expression is increased in astrocytes in response to inflammation and iron overload (Routhe et al. 2020) (Fig. 3d).

Iron plays an important role in the synthesis of myelin by oligodendrocytes (Benkovic and Connor 1993; Todorich et al. 2011; Belaidi and Bush 2016), and iron deficiency can cause myelination and other related diseases (Todorich et al. 2009). Oligodendrocytes may derive their iron from ferritin (Connor et al. 1995; Sanyal et al. 1996; Qi et al. 2002; Zhang et al. 2006; Todorich et al. 2011) and may take up iron primarily through endocytosis of ferritin (Masaldan et al. 2019). Age is correlated with the amount of iron in oligodendrocytes (Benkovic and Connor 1993). Also, Tf and FPN may, respectively, mediate the intracellular transit of iron and its efflux from oligodendrocytes (Moos et al. 2007).

Microglia are formed by the differentiation of circulating monocytes after they migrate to the brain (Milligan et al. 1991). During this process, iron and ferritin are released (Moos 1995; Cheepsunthorn et al. 1998) and may provide iron to oligodendrocytes (Todorich et al. 2011).

Like BMECs, iron is capable of reaching neurons by internalizing the Tf–TfR1 complex, where it is then reduced and delivered into the cytosol by DMT1 (Burdo et al. 2001). Interestingly, cellular prion protein (PrPc), which, like TfR1, has extensive expression on the outer layers of neurons forms a complex with DMT1 (Giometto et al. 1990), where DMT1 transports non-transferrin-bound iron (NTBI) into neurons and PrPc reduces Fe^3+^ to Fe^2+^ (Singh 2014; Tripathi et al. 2015). Similarly, excess iron efflux in neurons is mediated by FPN1, while Cp and hephaestin (Heph) promote the efflux of FPN and the oxidation of Fe^2+^ (Qian et al. 2007). Additionally, amyloid precursor protein (APP) and tau are key proteins involved in iron efflux in neurons (McCarthy et al. 2014; Belaidi et al. 2018). With the contribution of soluble tau, APP is transported to the cell membrane to stabilize FPN1 and promote iron export (Lei et al. 2012; Wong et al. 2014b) (Fig. 3d).

## The antioxidant system

In the face of oxidative damage caused by iron overload, intracellular antioxidant systems prevent cell death due to lipid peroxidation. Interestingly, ferroptosis, a novel cell death phenotype initially induced by Ras and ST eradicator (erastin) and Ras selective lethal 3 (RSL3) (Dixon et al. 2012), results from disruption of intracellular redox homeostasis precisely through depletion of GSH.

### GSH–GPX4 axis

Among them, the target of elastin, System Xc^−^ cystine/glutamate antiporter is inhibited by it to induce ferroptosis (Dixon et al. 2012, 2014). System Xc^−^ is a heterodimeric amino acid transport complex consisting of a heavy-chain 4F2hc protein (i.e., recombinant solute carrier family 3 member 2, SLC3A2) and a light-chain xCT protein (recombinant solute carrier family 7 member 11, SLC7A11) (Sato et al. 1999; Liu et al. 2021) (Table 1). SLC7A11 is a transport protein and a functional component of System Xc^−^, and its 12 putative transmembrane domains ensure the specificity of System Xc^−^. SLC3A2 is a single transmembrane regulatory protein responsible for maintaining the structural stability of System Xc^−^ (Koppula et al. 2018). System Xc^−^ is ubiquitously expressed in the brain, involving the blood–brain barrier and the entire brain parenchyma (Lewerenz and Maher 2011). Under oxidative conditions, in a 1:1 ratio, system Xc- facilitates the transfer of glutamate from the cell via an ATP-dependent mechanism in exchange for extracellular cystine (cysteine dimer cystine, Cys_2_) (Maher 2005; Cao and Dixon 2016). This process is regarded as the initial and crucial event in the induction of ferroptosis (Ou et al. 2022). Selenium-containing thioredoxin reductase (TXNRD1) rapidly reduces ingested cystine to cysteine, an essential substrate for the synthesis of GSH at the expense of NAD(P)H (Ursini and Maiorino 2020). Then, cysteine combines with glutamic acid and glycine to form GSH, and glutamate-cysteine ligase (GCL) and glutathione synthetase (GSS) are rate-limiting enzymes synthesized in these two steps, respectively (Seibt et al. 2019; Yan et al. 2022). Butythionine-(S,R)-sulfoxideimine (BSO) can induce ferroptosis by inhibiting GCL (Friedmann Angeli et al. 2014), while ferroptosis can be inhibited by α-tocopherol and DFO (Seiler et al. 2008). Among them, the pentose phosphate pathway is primarily responsible for producing NADPH. Studies have pointed out that malate 1 (ME1) reduces NADP^+^ to NADPH via the pentose phosphate pathway, which then affects the cystine–GSH–GPX4 antioxidant axis driven by it, thereby inhibiting ferroptosis (Fang et al. 1970). The pentose phosphate pathway's oxidative branch provides a significant amount of NADPH (Lane et al. 2021). (Fig. 3d).

The antioxidant effect of GSH is dependent on the reduction-active thiol groups on its cysteine residues (Dodson et al. 2019a). Therefore, GSH depletion by GPX4 reduces LOOH (e.g., PE-AA-OOH) to nontoxic lipid alcohols (LOH, e.g., PE-AA-OH) (Yang et al. 2014). GSSG generated by the oxidation of GSH is re-reduced to GSH under the catalysis of GSSG reductase (GSR), and NAD(P)H acts as a cofactor of GSR (Wu et al. 2011; Ammal Kaidery et al. 2019) (Fig. 3d).

### FSP1–ubiquinone (CoQ_10_) axis

Interestingly, some cancer cell lines did not undergo ferroptosis in the presence of GPX4 inactivation, suggesting the existence of other anti-ferroptotic mechanisms (Viswanathan et al. 2017), and the FSP1–NADH–CoQ_10_ axis is one of them, paralleling the GPX4 pathway play a role (Bersuker et al. 2019; Doll et al. 2019). FSP1, also known as AIFM2/AMID/PRG3), is a newly discovered antagonist of ferroptosis (Cheng et al. 2023). FSP1, which is located in the mitochondria, undergoes myristoylation and is subsequently recruited into the plasma membrane. This recruitment leads to a conversion of its pro-apoptosis activity into antiferrotic effects (Bersuker et al. 2019; Doll et al. 2019). The mechanism behind this conversion is believed to involve FSP1 acting as an NAD(P)H-dependent coenzyme Q_10_ (also known as CoQ_10_ or ubiquinone) oxidoreductase at the plasma membrane. In this role, FSP1 reduces CoQ_10_ to its antioxidant form, ubiquinol (CoQ_10_H_2_), which then scavenges lipid peroxyl radicals (Bersuker et al. 2019; Jiang et al. 2021), and ubiquinone is synthesized via the mevalonate pathway. Or FSP1 promotes oxidative damage repair of plasma membranes (Dai et al. 2020b). In addition, FSP1 can also act as an NAD(P)H-dependent vitamin K reductase, mediating non-canonical vitamin K cycling to protect cells from harmful lipid peroxidation and ferroptosis (Mishima et al. 2022) (Fig. 3d).

### The GCH1–BH_4_–phospholipid axis

GTP cyclohydrolase 1 (GCH1), a key enzyme in the endogenous synthesis of tetrahydrodiphosphate (BH_4_), is an additional ferroptosis inhibitor independent of GPX4 (Kraft et al. 2020). Synthetic BH_4_ is hypothesized to prevent ferroptosis by promoting the FSP1–CoQ_10_ axis to increase panthenol and regulating lipid peroxidation (Kraft et al. 2020; Soula et al. 2020) (Fig. 3d).

## Lipid peroxidation

Although iron overload is not a necessary condition for cellular ferroptosis, it is more common for it to cause dysregulation of LIP resulting in disturbance of the intracellular redox state. LIP contains unbound iron, and its alteration is caused by: 1. Induction of ferritin autophagy, leading to the release of Fe^2+^ by erastin or lack of cysteine; 2. Catalysis of heme decomposition by heme oxygenase 1 (HO1/HMOX1) to produce Fe^2+^; 3. Mediated uptake of ferritin-bound iron by Tf–TfR1; 4. Inhibition of iron storage or iron efflux (Kakhlon and Cabantchik 2002). The process of ferroptosis, also known as labile iron(Fe)-mediated "autooxidative" non-enzymatic lipid peroxidation, is initiated when there is an excess of active unbound iron, which can either directly accelerate the creation of free radicals or create ROS through the Fenton reaction (Brown et al. 2018). However, the exact mechanism of ferroptosis remains unclear (Feng and Stockwell 2018). The accumulation of active species such as ROS will cause lipid peroxidation, and the accumulation of its products and reactive degradation products will result in ferroptosis.

Thus, the build-up of LOOH is considered an important feature of iron death, and PUFA on phospholipids is a determinant of this due to preferential oxidation (Ou et al. 2022). As a result, the PUFA transport and binding proteins involved affect how sensitive the cell membrane is to phospholipid peroxidation events (Maiorino et al. 2018; Miotto et al. 2020). Acyl-CoA synthetase long-chain family member 4 (ACSL4) and lysophosphatidylcholine acyltransferase 3 (LPCAT3) are to be accountable for activation of PUFAs to produce long-chain lipid acyl-CoA (PUFA-CoA) (Table 1), insertion of acyl groups into lysophospholipids (PUFA-PE), respectively (Yuan et al. 2016; Doll et al. 2017). Further, lipid peroxidation leads to fragmentation of PUFAs and membrane lipid damage, leading to irreversible membrane perforation and ferroptosis through a transmissible osmotic mechanism (Riegman et al. 2020). Recent studies have identified PKCβII as an important sensor of lipid peroxidation. PKCβII senses initial lipid peroxidation and amplifies it through phosphorylation and activation of ACSL4 (Zhang et al. 2022a). H_2_O_2_ produced by NADPH-cytochrome P450 reductase generates free radicals catalyzed by Fe^2+^ (Zhang et al. 2022a). There are also lipoxygenases (LOX) containing oxidized trivalent non-heme iron that can directly oxidize PUFA on the membrane (PUFA-PE) (Wenzel et al. 2017; Chu et al. 2019; Stockwell and Jiang 2020; Yan et al. 2021a) (Fig. 3d). Reactive lipids (RLS) generated by the peroxidation of lipids, such as malondialdehyde, or MDA, and 4-hydroxy-2-nonenal (4HNE), have been linked to the development of several disorders, such as diabetes and neurodegeneration (Ayala et al. 2014).

## Nrf2

**Fig. 2:**
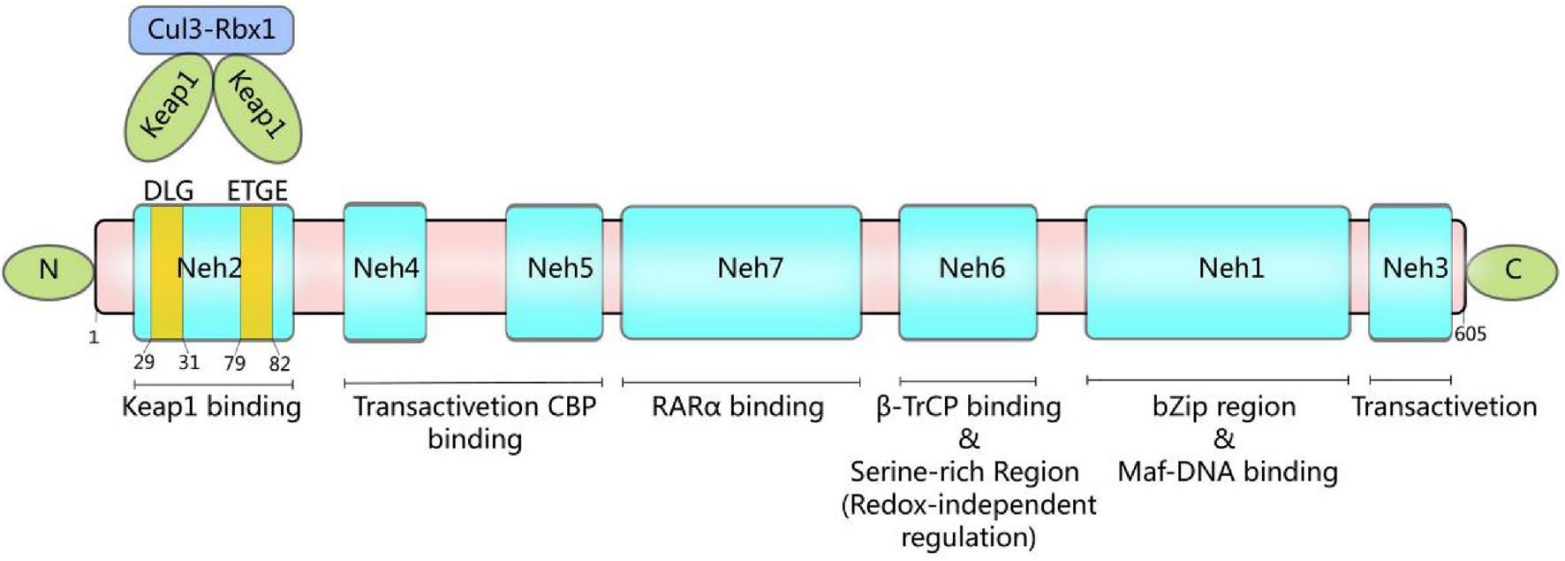
Nrf2 domain schematic depiction. Nrf2 comprises seven functional domains and 605 amino acid residues (Neh1–7). Neh2 located at the N-terminus has ^29^DLG^31^ and ^79^ETGE^82^ motifs, which can bind to the Keap1 homodimer of the E3-ubiquitin ligase complex Keap1–Cul3–Rbx1 to mediate its degradation. Neh4 and Neh5 bind to CREB-binding protein (CBP) and activate transcription. Neh7 binds to retinoic acid receptor alpha (RARα). Neh6 is rich in serine residues and inhibits Nrf2 by interacting with B transducin repeat-containing protein (B-TrCP). Neh1 contains a bZip motif, which can form a dimer with Maf or Bach protein, and then bind to DNA (specifically, the ARE sequences in the target gene promoter). Neh3 is the transactivation domain. Drawing by Inkscape

Nuclear factor E2-related factor 2 (Nrf2, encoded by the *Nfe2l2* gene) is a key transcription element involved in the control of intracellular oxidative stress (OS), directly regulating more than 200 antioxidant and phase II genes (Dodson et al. 2019b; Song and Long 2020), many of its downstream target genes are involved in ferroptosis, including FTL/FTH/FPN related to iron metabolism and GPX4/SLC7A11/GCL/GSH related to antioxidant system (Dodson et al. 2019a). Nrf2, along with Nrf1, Nrf3, the NF-E2 p45 subunit and the less related factors BTB domain and CNC homologs 1 and 2 (Bach1 and Bach2), belongs to the Cap 'n' Collar (CNC)-bZIP (basic leucine zipper) family of transcription factors (Toki et al. 1997; Itoh et al. 1999). This classification is based on the high homology of Nrf2 with its cross-species orthologs, such as SKN-1 in Caenorhabditis elegans and CncC from Drosophila melanogaster (Itoh et al. 1999), divided into seven structures from Neh1 to Neh7 (Sivandzade et al. 2019) (Fig. 2). The Neh1 domain is located at the C-terminal side of the molecule, is responsible for DNA binding (Sun et al. 2009) and contains the nuclear localization signal (NLS) (Theodore et al. 2008). The Neh2 domain at the N-terminal end of Nrf2 contains two Kelch-like ECH-associated protein-1 (Keap1) binding sites (ETGE and DLG) that interact with Keap1 to negatively regulate the Nrf2 signaling pathway (Suzuki et al. 2013). Neh3 is located at the C-terminal end of the molecule and is responsible for activating the antioxidant response element (ARE) in the target gene promoter to cis-regulate the target gene (Nioi et al. 2005). Neh4 and Neh5 are involved in transactivation to enhance the transcriptional activity of Nrf2 by recruiting the "cAMP (cyclic adenosine monophosphate) response element-binding" (CREB) proteins (Kwok et al. 1994; Katoh et al. 2001; Nioi et al. 2005). Neh6, a serine-rich residue, associates with Neh2 to mediate Nrf2 ubiquitination (Chowdhry et al. 2013). The Neh7 domain, discovered later, reduces the expression of Nrf2 target genes by binding to retinoic acid receptor α (RARα) and destroying the binding of CBP (CREB-binding protein) to Neh4 and Neh5 domains, so RARα is considered a repressor of Nrf2 (Wang et al. 2013).

Under physiological conditions, Nrf2 maintains a low level in the cytoplasm, and its half-life is very short (He et al. 2020). Due to different E3-ubiquitin ligase complexes [Keap1–Cullin 3–Ring box 1 (Keap1–Cul3–Rbx1) (He et al. 2020), S-phase kinase-associated protein 1-Cullin1-Rbx1/β-transducin repeat protein (SCF/β-TrCP) (Rada et al. 2011; Chowdhry et al. 2013; Cuadrado 2015), synoviolin/Hrd1 (Wu et al. 2014) and WDR23/DDB1 and Cullin4 associated factor 11 (WDR23/DCAF11) (Lo et al. 2017)], Nrf2 undergoes ubiquitination and degradation. Specifically, the two Keap1 molecules bind to ETGE and DLG located in the Neh2 domain of Nrf2 and recruit the Cul3–Rbx1 complex to maintain normal levels of Nrf2 (Kobayashi et al. 2004; Cullinan et al. 2004; Zhang et al. 2004); The interaction of β-TrCP and Skp1–Cul1–Rbx1 complex on the Neh6 domain phosphorylated by GSK-3β can also lead to the ubiquitination and degradation of Nrf2 (Rada et al. 2011; Chowdhry et al. 2013; Cuadrado 2015); The combination of DCAF11, DDB1–Cul4–Roc1 and Neh2 (Lo et al. 2017), the upregulation of Hrd1 caused by ER stress, can also mediate the degradation of Nrf2 (Wu et al. 2014). When cells are exposed to OS, gene mutations and other factors, the degradation of Nrf2 by the above complex may be impaired, such as: pro-oxidant conditions, autophagy disorders inhibit the combination of Keap1 and Nrf2 (Jain et al. 2010; Llanos-González et al. 2020), to activate Keap1–Nrf2–ARE axis (Sova and Saso 2018; Panieri and Saso 2019).

### Keap1–Nrf2–ARE axis

Keap1, which senses OS through cysteine residues, undergoes conformational changes and releases Nrf2 (Miseta and Csutora 2000). Among them, nucleophilic thiols including Keap1 cysteine sulfhydryl groups can be covalently bound to a variety of electrophile Nrf2 inducers (Prestera et al. 1993; Dinkova-Kostova et al. 2002), and this cysteine modification can sense electrophilic or oxidative conditions to activate Nrf2 (Holland et al. 2008). Keap1 can be poly-ubiquitinated by modifying its own central linker domain under specific chemical and OS conditions (Zhang et al. 2005a; Hong et al. 2005). Under stress conditions, binding of p62 to Keap1 (Hancock et al. 2012) drives the binding of microtubule-associated protein 1 A/1 B-light chain 3 (LC3), which in turn leads to Keap1 degradation (Komatsu et al. 2010). Nrf2 in turn upregulates the expression of the *p62* gene by binding to its ARE sequence, thereby forming a positive feedback loop (Jain et al. 2010). Due to the suppression of its breakdown brought on by these factors, Nrf2 levels in the cytoplasm rise, increasing its translocation to the cell nucleus. (Kobayashi and Yamamoto 2005; Zhang 2006).

**Fig. 3:**
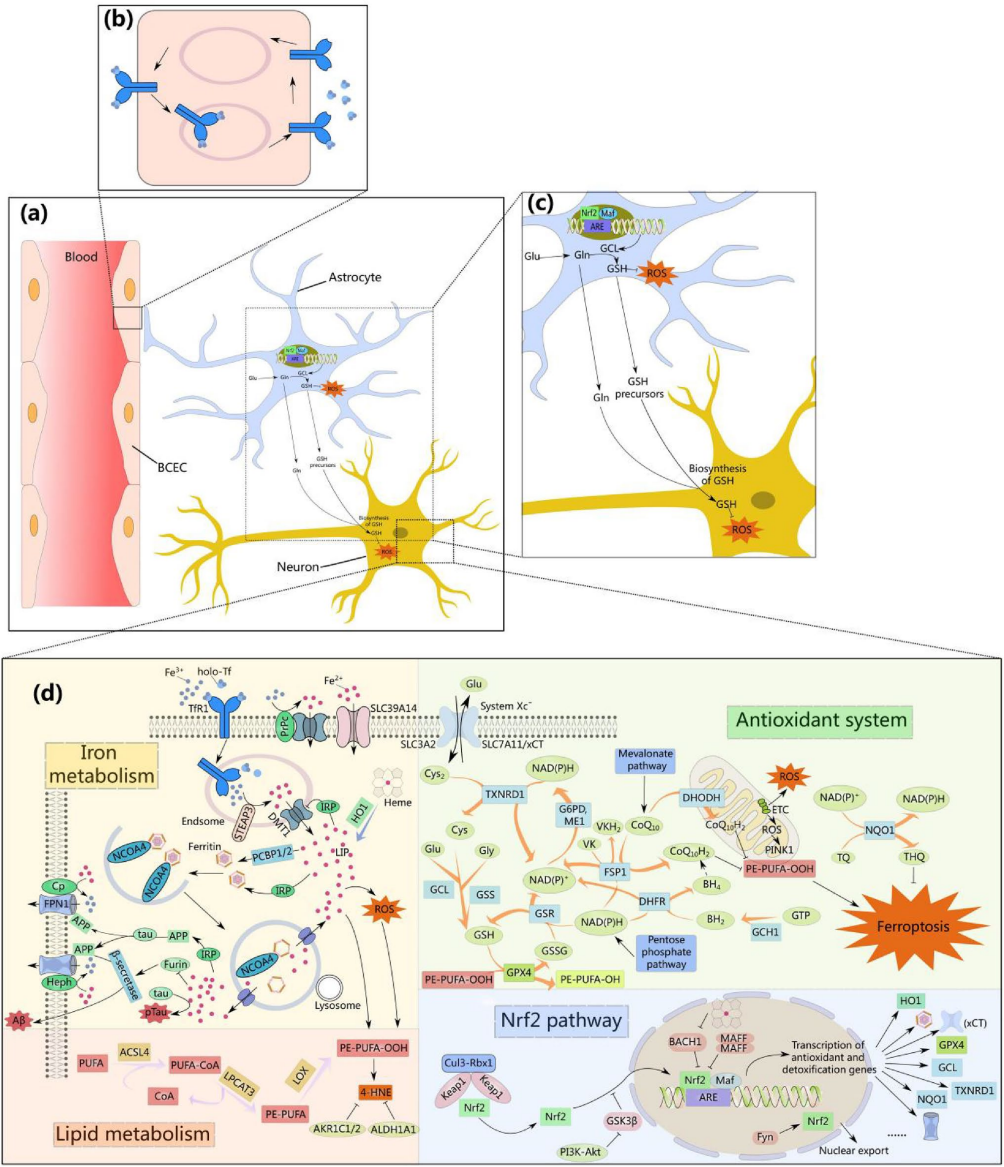
Nrf2-regulated iron death mechanisms in neurodegenerative diseases. **a** Blood–brain barrier (BBB), astrocytes, and neurons. Brain capillary endothelial cells (BCECs) safeguard the function of the BBB. Astrocytes on the abluminal surface of BCECs promote brain iron uptake. Astrocytes are primarily responsible for releasing iron into neurons while attenuating iron toxicity.** b** the transcytosis model. After Fe^3+^ entering the blood circulation forms a complex (holo-Tf) with transferrin (Tf), it binds to TfR1 on the surface of brain microvascular endothelial cells (BMECs), and then the Tf–TfR1 complex enters BMECs through clathrin-mediated endocytosis. One of the two models of iron import across BCEC—the transcytosis model. Holo-Tf is transported directly by vesicles to extraluminal sites for release into the brain.** c** Nrf2 in astrocytes activates non-cell-autonomous protection of nearby neurons and alleviates oxidative stress (OS) by mediating antioxidant responses. GSH produced in the brain via activated Nrf2 pathway is predominantly derived from astrocytes, whose neuroprotective function depends on the transport of GSH precursors from astrocytes to motor neurons. **d** Regulation of iron in neurons and mechanisms of Nrf2-regulated ferroptosis in neurodegenerative diseases. The key components of ferroptosis are the lipid and antioxidant systems, as well as iron metabolism. metabolism of iron: The Tf–TfR1 complex carrying Fe^3+^ is endocytosed into neurons, Fe^3+^ is separated from Tf, and then reduced to Fe^2+^ by six transmembrane epithelial antigen-prostate 3 (STEAP3), and Fe^2+^ is pumped into the cytoplasm through divalent metal transporter 1 (DMT1), which is also a classic model of iron import across BCEC. Alternatively, Fe^3+^ reduced by cellular prion protein (PrPC) is transported into neurons by DMT1. In addition, solute carrier family 39 member 14 (SLC39A14) can also transport Fe^2+^ into the cell. Intracellular iron can be stored in two forms: as Fe^3+^ stored in ferritin or as active unbound iron called the labile iron pool (LIP). Poly-(rC)-binding protein1/2 (PCBP1/2) are in charge of transferring the iron to ferritin. In some cases, nuclear receptor coactivator 4 (NCOA4) mediates ferritin autophagy, releasing iron. Heme oxygenase 1 (HO1) catalyzes the degradation of heme and can also release Fe^2+^. With the aid of amyloid precursor protein (APP), which is transmitted by soluble tau protein to stabilize FPN1, elevated Fe^2+^ can be expelled through Ferroportin1 (FPN1)/copper cyanine (Cp) or FPN1/hephaestin (Heph). Through the IRP–IRE interaction, iron can, when overloaded, increase the expression of ferritin, FPN1, and APP, while blocking the ordinary function of furin, upregulating secretase, and speeding up the deposition of Aβ. By the Fenton reaction, excessive Fe^2+^ produces ROS and encourages the oxidation of PUFA on the membranes of cells (PE-PUFA), and finally triggers ferroptosis. Lipid metabolism: Lysophospholipid acyltransferase 3 (LPCAT3), long-chain fatty acid CoA ligase 4 (ACSL4), and other enzyme-sensitize membrane lipids to lipid peroxidation, which, catalyzed by lipoxygenases (LOX), accumulates PUFA-OOH, triggering ferroptosis. Antioxidant system: GSH–GPX4 axis in cytoplasm and mitochondria, ferroptosis suppressor protein 1 (FSP1)–CoQ10 axis in plasma membrane, dihydroorotate dehydrogenase (DHODH)–CoQ_10_H_2_, and GTP cyclohydrolase1–tetrahydrobiopterin (GCH1–BH_4_) axis in mitochondria. Mitochondrial PTEN-inducible putative kinase 1 (PINK1) expression is regulated by Nrf2 under OS conditions. Keap1–Nrf2–ARE axis: Under OS conditions, Keap1 releases Nrf2, and the increase of Nrf2 levels in the cytoplasm increases its nuclear translocation. Upon nuclear import, Nrf2 forms a heterodimer with the small muscular aponeurotic fibrosarcoma (Maf) protein, enabling Nrf2 to bind to AREs in the upstream promoter regions of a variety of target genes, leading to their transcriptional activation. Drawing by Inkscape

Nrf2, after translocating into the nucleus, forms a heterodimer with small musculoaponeurotic fibrosarcoma (Maf) proteins (in vertebrates, MafF, MafG, or MafK), specifically in the CNC-bZIP region of the Neh1 domain. This heterodimerization enables Nrf2 to bind to AREs in the upstream promoter regions of various target genes, thereby leading to their transcriptional activation (Motohashi et al. 2004; Hirotsu et al. 2012) (Fig. 3d). Expression of these target genes protects cells from ferroptosis due to lipid peroxidation by participating in the regulation of iron metabolism and antioxidant systems. Interestingly, as a key regulator of ferroptosis, Nrf2 not only has a wide range of regulation, but also has a strong regulation ability. The promoter of its gene contains two ARE-like sequences, so that the downstream target genes have a longer expression time (Kensler et al. 2007). Nrf2 plays a key role in mediating iron/heme metabolism by regulating genes such as FTH1, FTL and FPN1 (Chen et al. 2020).

### FTH/FTL

FTH1 (ferritin heavy chain 1) and FTL (ferritin light chain) are the two subunits that make up ferritin. Among them, FTH1 can oxidize Fe^2+^ to Fe^3+^, and FTL promotes the entry of free iron into the ferritin mineral core (Chen et al. 2020).

### FPN1

Ferroportin1 (FPN1, additionally referred to as solute carrier family 40 member 1, SLC40A1) is currently the sole known cellular exporter of non-heme iron (Drakesmith et al. 2015), widely distributed in the CNS (Smith et al. 1997), and is thought to have a significant regulation influence on the iron metabolism of the CNS (Kalinina et al. 2014; Ma et al. 2020; Bao et al. 2020, 2021), which is modulated by Nrf2 transcription (Marro et al. 2010). Knockdown of Nrf2, which upregulates FPN1, prevents BMECs from transporting iron to the brain via FPN1 (Chen et al. 2020; Han et al. 2021), so it may be possible to lower Nrf2 to prevent brain iron excess (Han et al. 2021). It should be pointed out that the majority of the target genes mediated by Nrf2 function as antioxidants (Hayes and Dinkova-Kostova 2014).

FPN1 is also inhibited by hepcidin (Nemeth et al. 2004; Billesbølle et al. 2020). Hepcidin is secreted peripherally by the liver (Ganz 2013) and readily crosses the BBB. There may be a strong correlation between decreased FPN1 expression on the plasma membrane of neurons and particular kinds of glial cells (Devos et al. 2020), but the link between hepcidin levels in the brain and changes in iron levels in PD or ALS remains unclear (Nemeth et al. 2004; Jeong and David 2006).

### HO1

Heme oxygenase 1 (HO1, HMOX1), responsible for catalyzing the heme's breakdown to yield equimolar levels of Fe^2+^, biliverdin, and CO (Loboda et al. 2016) (Fig. 3d). The antioxidant properties of biliverdin and its reduced product bilirubin [mediated by biliverdin reductase (BVR)] can inhibit ferroptosis, while the redox activity of Fe^2+^ promotes (Loboda et al. 2016). Therefore, the regulation of HO1 on ferroptosis is two sided and depends on cell type and background (Sun et al. 2016; Chen et al. 2020). In HT1080 fibrosarcoma cells, HO1 overexpression facilitated ferroptosis brought on by erastin (Kwon et al. 2015; Chang et al. 2018); however, knockdown of HO1 produced the same effect in renal proximal tubular cells and hepatoma cells (Sun et al. 2016; Adedoyin et al. 2018). The regulation of ferroptosis by HO1 can be influenced by a combination of HO1-catalyzed heme metabolites and other Nrf2 target proteins, including FTH1, FTL, FPN1, and BVR (Lane et al. 2021).

### NQO1

NAD(P)H:quinone oxidoreductase 1 (NQO1), a classical Nrf2-regulated NAD(P)H-dependent dehydrogenase (quinone) reductase (Ross and Siegel 2017), inhibits ferroptosis (Sun et al. 2016). Using two electrons, NQO1 can effectively catalyze the conversion of ubiquinone, a quinone, to hydroquinone (Ernster 1967; Hosoda et al. 1974; Lind et al. 1982; Thor et al. 1982), and also act as a superoxide reductase to exert antioxidant effects (Siegel et al. 2004; Zhu et al. 2007). In addition, vitamin E quinone (α-tocopheryl quinone, TQ), as an endogenous quinone substrate for NQO1, can be reduced to the potent antioxidant α-tocopheryl hydroquinone (THQ) to maintain its antioxidant activity (Siegel et al. 1997), and act as a potent endogenous lipid peroxidation and ferroptosis inhibitors (Hinman et al. 2018) (Fig. 3d). NQO1 is upregulated through Nrf2 in response to OS, and elevated NQO1 associated with AD pathology is often viewed as a neuroprotective response to OS in AD (Raina et al. 1999; Wang et al. 2000; SantaCruz et al. 2004). NQO1 may also cooperate with FSP1 to exert anti-ferroptosis effect (Santoro 2020). Sensitivity of U2OS cells to RSL3 was unaffected in the absence of NQO1, however, compared to cells lacking only FSP1, cells lacking both FSP1 and NQO1 were far more sensitive (Bersuker et al. 2019).

### Antioxidation system

In the antioxidant system, the target enzymes of Nrf2 are involved in glutathione synthesis and metabolism, including GCLC and its modified subunits GCLM, GSS, xCT protein (SLC7A11), which are required for the synthesis of GSH (Ishii et al. 2000; Chan and Kwong 2000; Kwak et al. 2002; Sasaki et al. 2002; Yang et al. 2005), and maintain and increase GSH levels to ensure the ferroptosis suppression effect of GPX4. In particular, in mitochondria, dihydroorotate dehydrogenase (DHODH), which uses the oxidation of dihydroorotic acid to form orotate to mediate the reduction of ubiquinone to keep ubiquinone levels in mitochondrial membranes, is also regulated by Nrf2 (Mao et al. 2021). Among the intermediate metabolites regulated by Nrf2 is the aldoketone reductase (AKR) class (Penning 2017). AKR reduces aldehydes and ketones to alcohol forms at the expense of NAD(P)H consumption (Penning 2017). 4-Hydroxynonenal (4-HNE), an outcome of the lipid peroxidation procedure (Jaganjac et al. 2020), which has cytotoxic effects such as damaging structural proteins, reducing enzyme activity, and inducing cell death (Craighead 1995), is significantly elevated in ferroptosis (Feng and Stockwell 2018). Elevated 4-HNE is found in the cerebral tissues of individuals with Alzheimer's after autopsy (Yoo et al. 2010). Possibly, 4-HNE attacks amyloid-β (Aβ) peptides and forms adducts that encourage the production of proto-fibrils and disease-associated accumulation (Siegel et al. 2007). In reaction to Nrf2 activators, AKR1C1 and AKR1C2 are continuously excessively expressed and detoxify by decreasing 4-HNE (Penning 2017). Additionally, there are Nrf2 target enzymes such as aldehyde dehydrogenase 1 family member A1 (ALDH1A1), which oxidizes aldehydes to the carboxylic acid form, and glucose 6-phosphate dehydrogenase (G6PD), which is responsible for glucose metabolism/NAPDH neurodegeneration (Thimmulappa et al. 2002; Huang et al. 2010) (Fig. 3d).

### Regulation of Nrf2

As previously mentioned, in addition to Nrf2 negative regulators such as Keap1 and GSK3β, BTB domain and CNC homolog 1 (*BACH1*), a heme-binding transcriptional repressor of Nrf2-regulated genes (Dhakshinamoorthy et al. 2005), is stable under physiological conditions and interacts with Nrf2 Competes binding to ARE sequences to repress Nrf2 downstream target genes (Warnatz et al. 2011). In response to OS and/or elevated heme levels, *BACH1* is inactivated, leading to Nrf2-induced transcriptional activation (Ogawa 2001; Suzuki et al. 2003; Sun et al. 2004). For example, HO1 expression is repressed by *BACH1* at low levels of labile heme; however, an increase in labile heme causes *BACH1* to degrade and thus relieves this repression (Zhang et al. 2018). The study found that for erastin-induced GLCM, xCT, FTH1, FTL, and FPN1, and other antioxidant system and iron metabolism-related proteins, *BACH1* inhibited the transcription of their coding genes and promoted ferroptosis (Nishizawa et al. 2020). Activated GSK3β can also phosphorylate Fyn kinase (a member of the Src family), leading to its nuclear accumulation while Nrf2 nuclear export (Jain and Jaiswal 2007). Nrf2 can be activated by negative regulation of GSK3β, such as the activated PI3K–Akt pathway (Chowdhry et al. 2013), and AMP-activated protein kinase (AMPK). AMPK can also directly phosphorylate Nrf2 to promote its nuclear accumulation (Joo et al. 2016) (Fig. 3d).

## AD

AD is a neurodegenerative disease with a complex pathological mechanism that mainly damages cortical and hippocampal neurons, characterized by senile plaques (Cheignon et al. 2018) and neurofibrillary tangles (NFT) (Wan et al. 2019). Senile plaques are derived from aggregated insoluble amyloid beta (Aβ), while intracellular NFT are formed from hyperphosphorylated microtubule-associated protein tau (Masaldan et al. 2019). However, the pathogenesis of AD cannot be fully explained by the aggregation of Aβ and tau proteins alone, and strategies to reduce the burden of Aβ have not been effective in limiting disease progression (Morris et al. 2014). Intracellular iron deposition is present and associated with these pathological features long before development (Goodman 1953; Connor et al. 1992; Smith et al. 1997, 2007; Lovell et al. 1998), Ferroptosis has been recognized as a significant factor in the death of neurons in patients with AD (Ashraf et al. 2020; Park et al. 2021). Abnormal iron elevations are more significant in the preclinical stages of AD, especially in the AD-affected cortex and hippocampus (Smith et al. 1997; Belaidi and Bush 2016). It has been proposed that the rate of cognitive deterioration brought on by A may be predicted by the amount of iron that accumulates in the hippocampus (Ayton et al. 2017). It is widely acknowledged that iron has a role in the early pathophysiology of AD (Goodman 1953).

When iron overloads, hypoxia-inducing factor α (HIFα) is degraded and down-regulates iron-regulatory protein (IRP), which binds to iron-regulatory elements (IRE) linked to iron metabolism in brain tissue, inhibits the translation of TfR1 and DMT1 and promotes the translation of ferritin, FPN1, and APP (Rogers et al. 2002; Anderson and Frazer 2017), which leads to increased iron storage and iron outflow in neurons and decreased iron intake (Meyron-Holtz et al. 2004). At this time, the balance of APP lyases called α- and β-secretases regulated by furin is disrupted, and together with the upregulation of APP, Aβ deposition is accelerated (Silvestri and Camaschella 2008; Guillemot et al. 2013). Abnormally cleaved APP no longer assists FPN1, leading to inhibition of iron export and aggravated iron accumulation (Ward et al. 2014; Greenough 2016). At elevated extracellular iron levels, soluble Aβ binds to Fe^3+^ and promotes its reduction to Fe^2+^, which is difficult to dissociate, and additional senile plaques are created as a result of the rapidly precipitation of Aβ caused by the ROS produced in this phase (Ha et al. 2007) (Fig. 3d). However, there are multiple possible pathways for iron-induced tau hyperphosphorylation in neurons: the palliative effect of insulin suggests interference with insulin signaling as a possible cause (Wan et al. 2019); iron-mediated activation of the cyclin-dependent kinase 5 (CDK5)/P25 complex and glucose Prosynthase kinase 3β (GSK3β) may also be involved (Guo et al. 2013a); excess intracellular Fe^2+^-induced oxygen free radical production can also be induced by activation of extracellular signal-regulated kinase 1/2 (Erk1/2) or mitogen-activated protein Kinase (MAPK) signaling pathway plays the same role (Muñoz et al. 2006; Chan and Shea 2006). In turn, the reduction of soluble tau protein levels in brains with AD inhibits FPN1 activity, exacerbating brain iron deposition (Lei et al. 2012). The participation of activated glial cells in the development of neuroinflammation is a distinctive feature of the pathology of AD (Newcombe et al. 2018; Leng and Edison 2021). Microglia activated by increased brain iron levels, possibly via pro-inflammatory cytokines that are mediated by nuclear factor-B (NF-B) (Meng et al. 2017), overexpress ferritin to remove extracellular iron (Streit et al. 2022), triggering intracellular iron retention and TNFα expression increase (Holland et al. 2018; Kenkhuis et al. 2021), eventually leading to the formation of Aβ-plaques in the permeable zone (Peters et al. 2018; Kenkhuis et al. 2021). In addition, Lf secreted by activated microglia interacts with APP to promote Aβ formation (Tsatsanis et al. 2021). Similarly, the formation of Aβ induced increased expression of the pro-inflammatory factor IL-1β in microglia in a high-iron environment (Nnah et al. 2020). Astrocytes resist high iron-induced OS by significantly increasing GSH, catalase, and manganese superoxide dismutase (Iwata-Ichikawa et al. 2002; Shih et al. 2003). However, later findings overturned the previous understanding: when astrocytes are activated, they release inflammatory cytokines and trigger OS, which promotes the production of Aβ and tau tangles and impedes the clearance of Aβ (Cai et al. 2017). Metabolites such as 4-HNE and malondialdehyde produced by iron overload-mediated enzymatic (LOX) and/or non-enzymatic (Fenton reaction) lipid peroxidation are also elevated in AD brain (Wong et al. 2014a; Yang et al. 2016), then the PUFA on the membrane is depleted (Ansari and Scheff 2010; Elharram et al. 2017). In iron metabolism, to block iron entry into the brain, knockdown of Nrf2 reduces FPN expression on BMECs and may serve as a means to avoid brain iron overload (Han et al. 2021).

However, Nrf2 plays a critical role in the regulation of the antioxidant system. Age-related decline in Nrf2 levels, particularly in the brains of patients with AD (Osama et al. 2020), could potentially signify an early occurrence of ferroptosis (Habib et al. 2015). Although OS levels ARE elevated in AD, the transcriptional activation of Nrf2 is defective and/or incomplete due to reduced nuclear entry and/or defective binding to ARE (Kraft et al. 2004; Ramsey et al. 2007). As mentioned earlier, the excessive activation of GSK3β not only mediates the degradation of Nrf2, but also participates in tau hyperphosphorylation, Aβ aggregation-related neuroinflammation (Hooper et al. 2008), making it a possible reason for the decreased Nrf2 activity in AD (Lane et al. 2021). The negative regulation for Nrf2 by *BACH1* is another enabler (Di Domenico et al. 2015; Perluigi et al. 2020). In trisomy 21, *BACH1* located on chromosome 21 is elevated, the target gene of Nrf2 is suppressed, and confers an increased risk of AD-like dementia (Perluigi et al. 2020). Limited studies have demonstrated that *BACH1* changes occur in the AD brain (Shim et al. 2003). Interestingly, a meta-analysis showed that in the presence of increased *NRF2* gene expression, 31 target genes of Nrf2 were still significantly downregulated in AD (Wang et al. 2017). In contrast, the gene encoding the sMAF protein MafF is markedly increased in AD, which may partly explain the defect in Nrf2 stimulation (Wang et al. 2017): increased MarF promotes its homodimer formation, leading to repression of Nrf2 (Igarashi et al. 1994) (Fig. 3d).

The influence of lipid peroxidation on the Nrf2 pathway is twofold (Dinkova-Kostova et al. 2017): on the one hand, the electrophilic modification of Keap1 by lipid peroxidation products such as 4-HNE can activate the Nrf2 pathway; on the other hand, excessive lipid peroxidation leads to inactivation of Nrf2-regulated antioxidant-related enzymes such as GCL, TXNRD1, and PRDX6 (Dinkova-Kostova et al. 2017), which disrupts the homeostasis of the redox system and drives ferroptosis in AD (Dodson et al. 2019a). Similarly, with changes in Nrf2, its ferroptosis-related target proteins are altered in AD (Lane et al. 2021). In AD brain, the levels of GSH, GPX, GST, GR and other antioxidant enzymes decreased, while GSSG increased (Ansari and Scheff 2010). The study discovered a beneficial association between cognitive deterioration and decreased GSH expression in the cerebral cortex of AD animal models (Karelson et al. 2001). Consequently, the amount of GSH have been suggested to be predictive of AD and moderate cognitive impairment in the frontal brain lobe and hippocampus (Mandal et al. 2015; Ayton et al. 2020). Selenium levels affect the activity of GPX4 and TXNRD1, which have been reported to be inversely connected to cognitive deterioration (Cardoso et al. 2015). And the meta-analysis confirmed that the reduction in selenium content occurs in the affected temporal cortex of the AD brain (Cardoso et al. 2017; Varikasuvu et al. 2019). In addition to the regulation of transcriptional activation of the antioxidant system, Nrf2 is also regulated in AD in other ways (Bahn et al. 2019). BACE1 is primary enzyme accountable for the production of Aβ, which plays an important role in the pathogenesis of AD (Vassar et al. 1999; Cai et al. 2001; Selkoe 2012), and can bind to the long non-coding RNA (BACE1-AS) transcribed from its complementary strand to stabilize BACE1 mRNA (Faghihi et al. 2008). Increased BACE1-AS and BACE1 expression, Aβ deposition and cognitive deficits were more pronounced in Nrf2-deficient AD mice compared with Nrf2-deficient mice and AD mice, suggesting that Nrf2 is a negative regulator of BACE1-AS and BACE1 (Bahn et al. 2019). Nrf2 inducers, such as sulforaphane from cruciferous vegetables, and tert-butylhydroquinone (tBHQ) (Kraft et al. 2004), activate Nrf2 to inhibit the expression of BACE1 and BACE1-AS through the modification of the cysteine residue of Keap1 to prevent the production of Aβ, a key pathogenic process in the early stages of AD (Dinkova-Kostova et al. 2002; Kobayashi and Yamamoto 2006; Bahn et al. 2019). Nrf2 expression in adjacent astrocytes may determine the protective effect of Nrf2 inducers on neurons (Kraft et al. 2004). Nrf2 expression in neurons, although not as likely to be dominant as in glial cells (Kraft et al. 2004; Liddell 2017), is also believed to be crucial in the body’s defense against damage from oxidization (Ramsey et al. 2007). When Nrf2 is lacking, neurons are more vulnerable to cell death caused on by oxidative stressors such H_2_O_2_ (Kraft et al. 2004); conversely, overexpression of Nrf2 can protect neurons (Lee and Johnson 2004).

## PD

PD, also known as tremor paralysis, is a common motor system-specific neurodegenerative illness (Ammal Kaidery et al. 2019). The primary pathogenic features of PD are the persistent reduction of dopaminergic neurons containing neuromelanin in the substantia nigra densa (SNpc) and the formation of intracellular inclusion bodies by α-synuclein (α-Syn), i.e., Lewy bodies (Lee Mosley et al. 2006; Hustad and Aasly 2020). The following elements work together to cause PD: The biggest risk factor is age (Ammal Kaidery et al. 2019); additionally, it is believed that OS, mitochondrial malfunction, and neuroinflammation are the primary processes causing cell death in PD (Jenner and Olanow 2006; Yacoubian and Standaert 2009); meanwhile, environmental and genetic factors also affected it. In addition, abnormal iron deposits have been found in the brains of PD patients (Lhermitte et al. 1924). These factors interact with progressive PD, complicating the pathogenesis of the latter (Yang et al. 2022b).

An significant manifestation of OS is the increase of ROS and RNS levels, and mitochondria are an important source of ROS in cells (Di Meo et al. 2016). Immunohistochemical discovery of 4-HNE protein adducts provided early support for an etiological link of ROS with PD in postmortem neurons in the substantia nigra (SN) of the brain of seven PD patients (Yoritaka et al. 1996). The occurrence of OS in PD has also been demonstrated in animal models of PD (Wang et al. 2021a). As a byproduct of heroin synthesis, 1-methyl-4-phenyl-1,2,3,6- tetrahydropyridine (MPTP) has been shown to cause Parkinson-like symptoms (Langston et al. 1983; Dong et al. 2021). Further studies have found that MPTP induces cellular oxidative stress by indirectly affecting mitochondrial complex I (nicotinamide adenine dinucleotide (NADH) cytochrome c reductase) to block the electron transport chain (ETC), leading to cell death (Poirier and Barbeau 1985) (Fig. 3d).

Insecticides such as rotenone (Sharma et al. 2021), maneb, and paraquat (Ahmad et al. 2021) have similar mechanisms of action (Ammal Kaidery et al. 2019). Meta-analysis found an increased risk of developing PD after exposure to insecticides (Gunnarsson and Bodin 2019). Schapira's team is the first to identify a defect in PD brains' mitochondrial complex I, compared to other neuronal regions that are normal (Schapira et al. 1989, 1990). Further, autopsy revealed increased mutations and loss of mitochondrial DNA in tissues and blood cells of PD patients suggesting that the malfunctioning of the mitochondria is related to PD. (Coxhead et al. 2016; Pyle et al. 2016). There is mounting evidence that PD is characterized by malfunctioning mitochondria (Prasuhn et al. 2021). Mitochondrial metabolism provides brain cells with energy to maintain them survival (Nolfi-Donegan et al. 2020; Rose et al. 2020), while energy-storing ATP relies on mitochondrial respiration and electron transport chain (ETC) synthesis through oxidative phosphorylation. Among them, the complexes I–V that constitute the mitochondrial ETC are distributed in the inner mitochondrial membrane (Guo et al. 2017; Zhao et al. 2019). In PD, complex I deficiency is the main cause of ROS generation, while ROS are mainly generated in complex I and complex III (Kussmaul and Hirst 2006). Mitochondrial dysfunction produces excessive ROS, which can aggravate mitochondrial damage by attacking mitochondrial DNA, forming a vicious cycle (Elfawy and Das 2019). At the same time, damaged mitochondria can also affect energy metabolism, calcium homeostasis, and eventually induce apoptosis (Markesbery 1997; Peoples et al. 2019; Wang et al. 2021a). NADPH oxidase (NOX) is a class of ROS-producing enzymes, usually divided into seven isoforms: NOX 1 to 5 and dual oxidase (DUOX 1 to 2) (Bedard and Krause 2007). As multisubunit membrane-bound enzymes, they transfer electrons from NADPH to O_2_ to generate superoxide (Bedard and Krause 2007). Among them, Numerous areas of the brain and different types of brain cells express NOX2, including neurons in striatum (McCann et al. 2008; Guemez-Gamboa et al. 2011) and SN (Zawada et al. 2011; Qin et al. 2013), and compared with neurons and astrocytes, it has a higher expression in microglia More expressed, which was also confirmed in experimental models of PD and PD (Gao et al. 2002; Zhang et al. 2005b; Wu et al. 2005). Furthermore, in astrocytes, NOX4 is the major NOX isoform (Nayernia et al. 2014). Finally, dopaminergic neurons in the SN may also generate ROS through dopamine metabolism leading to neurodegenerative processes (Chinta and Andersen 2008; Hastings 2009).

In PD, OS occurrence is also associated with impaired antioxidation, which is supported by the reduction in GSH levels found in the SN of postmortem PD brains (Sofic et al. 1992), which also further increases iron content (Dexter et al. 1989), causing the formation of ROS and toxic effects on brain area (Kaur et al. 2003). In addition, significant increases in CoQ_10_ and 8-OHdG (nuclear DNA oxidation biomarkers) were also found in cerebrospinal fluid samples from PD patients (Isobe et al. 2010). Unlike GSH, HO1 levels are elevated in the SN of human PD brains (Schipper et al. 1998). NQO1, also a Nrf2 target enzyme, was also found to be increased in astrocytes, endothelial cells, and dopaminergic neurons in postmortem PD brains (Schipper et al. 1998). According to these postmortem results, Nrf2’s target genes are activated by enhanced nuclear import in PD brains (Fão et al. 2019).

Not only that, but there is also direct evidence that Nrf2 levels are increased in the nucleus of SN neurons in human PD patients (Ramsey et al. 2007). Nrf2 has antioxidant and neuroprotective effects (Yang et al. 2022b). H_2_O_2_ in the normal concentration range was found to mediate activation of the Nrf2 signaling pathway in glial cells, protecting dopaminergic neurons (Wang et al. 2021b). There is substantial evidence that Nrf2–ARE pathway activators show greater resistance to neurotoxins including MPTP, paraquat and rotenone in both in vivo and in vitro models (Wang et al. 2021a). Among the numerous target enzymes regulated by Nrf2, HO1 exerts neuroprotective effects against OS injury and may become a new therapeutic target for PD (Jazwa and Cuadrado 2010). Higher concentrations of HO1 are present in the serum of PD patients (Sun et al. 2021). In contrast, Downregulation of HO1 and Nrf2 causes α-synuclein aggregation (He et al. 2013). Presumption of HO1-induced neuroprotection against exposure to multiple PD-associated neurotoxins confirmed based on results from animal models and tissue culture (Kwon et al. 2019; Inose et al. 2020). A recent study found that the PI3K–AKT–glycogen synthase kinase 3 (GSK3) pathway is activated by the chemokine fractalkine (CX3CL1)–CX3CR1 axis to upregulate Nrf2 (Subbarayan et al. 2022), thereby protecting microglia proliferation from the deleterious effects of PD (Yang et al. 2022b). NF-κB, which induces the expression of multiple pro-inflammatory genes, was inhibited by Nrf2 (Cao et al. 2022). Nrf2 also regulates mitochondria-related genes, including the upregulation of the multifunctional DNA-binding protein mitochondrial transcription factor A (TFAM), which has a major impact on the expression of the mitochondrial genome, in addition to the transcriptional activation of essential function-related genes (such as NADH and NADHP formation-related enzymes, NOX) (Yang et al. 2022b). PTEN-inducible putative kinase 1 (PINK1) modulates mitochondrial quality and its expression is regulated by Nrf2 under OS conditions (Murata et al. 2015). Interestingly, PINK1 is not only associated with its stabilization on the dysfunctional mitochondrial outer membrane, but is also involved in the recruitment of p62 for mitochondrial removal (Geisler et al. 2010; Narendra et al. 2010). In a possible connection with this, an ARE element has been reported in the p62 promoter (Jain et al. 2010). Furthermore, p62 stabilizes and activates Nrf2 by interacting with Keap1, tumor suppressor genes (WTX), partner and localizer of BRCA2 (PALB2), dipeptidyl-peptidase 3 (DPP3) and CDK 20) (Komatsu et al. 2010; Fu et al. 2019). Consistent with this, it was found that Lewy bodies in the SN of early PD brains may be associated with p62 (Kuusisto et al. 2003), both in terms of oxidative damage shown by the *p62* gene promoter in the frontal cortex of PD brains and in terms of p62 proteasomal degradation (Du et al. 2009) due to mutations in the *parkin* gene that stabilizes p62 in PD (Lesage and Brice 2009; Song et al. 2016). In addition, PI3K/Akt pathways is inhibited in dopaminergic neurons of the human PD brains (Malagelada et al. 2008). NF-kB accumulates and translocates in the nucleus and increases (Hunot et al. 1997; Soós et al. 2004; Mogi et al. 2007). The above studies indicate that p62, PI3K–Akt pathway, and NF-kB may become potential targets for the treatment of PD (Fão et al. 2019).

## HD

Huntington's disease (HD) is a global autosomal dominant delayed ND (Ross and Tabrizi 2011). Mutant huntingtin (mtHtt), an important trigger and pathological feature of HD, is caused by abnormal CAG trinucleotide repeat in the huntingtin (*HTT*) gene, resulting in amplification of polyglutamine repeats in mtHtt (Tabrizi et al. 2019; Zgorzynska et al. 2021). The N-terminal truncated mtHtt is mismodified to form toxic soluble monomers or small oligomeric fragments with abnormal conformation and further synthesis of oligomers (Ou et al. 2022), precursors of fibrils in the cytoplasm and nucleus (DiFiglia et al. 1997; Cooper 1998; Hoffner et al. 2005). This leads to the dysfunction of the scaffolding protein Htt involved in various physiological processes such as vesicle trafficking, autophagy (Benn et al. 2008; Saudou and Humbert 2016), and eventually leads to degenerative diseases in the striatum and cortex areas of the brain (McColgan and Tabrizi 2018). Impairment of ubiquitin–proteasome (UPS) and autophagy systems affects protein degradation in HD animal models and human tissues (Ravikumar et al. 2004; Lin et al. 2013; Cortes and La Spada 2014). In addition, dysfunction of Htt also affects Brain-Derived Neurotrophic Factor (BDNF) production and inhibition of apoptotic factor activity (Gauthier et al. 2004; Zuccato and Cattaneo 2014; Saudou and Humbert 2016). These toxic macromolecules enter the nucleus of neuronal cells and bind and interact with DNA, while their amplified polyglutamine tracts cause transcriptional dysregulation of antioxidant genes in the binding areas (Hensman Moss et al. 2017; Ferrari Bardile et al. 2019; Tabrizi et al. 2020). These localized toxic fragments in cytoplasm and nucleus can damage mitochondria, resulting in reduced ATP, increased ROS, and other effects (Ayala-Peña 2013).

Compared with the complex pathological mechanism, OS, the initial onset of this HD is clear (Chen et al. 2007). Existing evidence suggests that mtHtt-activated microglia and astrocytes release cytokines that promote ROS production, with increased glutamine release from astrocytes but reduced BDNF from the same astrocytes (Ferguson et al. 2022). Specifically, mtHtt interacts with the inner and outer membranes of mitochondria, thereby blocking mitochondrial protein import (Yablonska et al. 2019), also determines cell death (Choo 2004). Lower levels of glucose metabolism and higher concentrations of lactate than normal in some regions of the HD brain (Reynolds et al. 2005; Tabrizi et al. 2020) corroborate findings of disrupted mitochondrial ultrastructure in the brain of an adolescent HD patients (Goebel et al. 1978). The selective striatal neurotoxin 3-nitropropionic acid (3-NP) can mimic the brain damage observed in HD patients by inducing OS (Brouillet et al. 2005) due to its inhibition of succinate dehydrogenase enzyme (complex II) (Damiano et al. 2010; Zuccato et al. 2010), further confirming the association between HD and mitochondria respiratory chain (Tucci et al. 2022). 3-NP is also involved in the activation of microglia and promotes the release of inflammatory cytokines that lead to neurological dysfunction and striatal neuronal death (Kim et al. 2017). Importantly, in HD, the interaction of mHTT and OS exhibited a prominent bidirectional line (Tobore 2019). OS also exerts an important influence on the mHtt accumulation in the nucleus, such as attacking the proteasome (Son et al. 2019), promoting mHtt aggregation (Goswami et al. 2006). Similarly, the mitochondrial NOX pathway and the Fe^2+^-involved Fenton reaction are sources of ROS (Tucci et al. 2022). Regarding the link between HD and NOX, high levels of NOX, mainly NOX2 subtypes, were found in the striatum of HD patients (Ibrahim and Abdel Rasheed 2022). Therefore, iron overload is an important factor in triggering HD (Agrawal et al. 2018). Assays in the R6/2 HD mouse model showed elevated ferritin levels in the striatum and cortex, decreased IRP and TfR, and increased FPN (Simmons et al. 2007; Chen et al. 2013). Iron supplementation to HD mice resulted in further shrinkage of the striatum and aggravation of neurodegeneration (Berggren et al. 2015). Conversely, intracerebroventricular injection of iron chelator (DFO) in R6/2 HD mice ameliorated striatal lesions (Agrawal et al. 2018). Similar positive effects were also observed in isolated HD rat brain slices, where treatment with the ferroptosis inhibitor Fer-1 and iron chelators significantly reduced neuronal death (Skouta et al. 2014). These evidences suggest a possible role for ferroptosis in HD (Ou et al. 2022). Autopsy revealed decreased plasma GSH levels (Klepac et al. 2007) and increased lipofuscin (suggesting dysregulation of lipid oxidation) in HD brains (Pérez-Severiano et al. 2000; Stoy et al. 2005; Browne and Beal 2006). Similarly, in the R6/2 HD mouse model, both mtHtt inclusions in striatal neurons and increased lipid peroxidation were found (Agrawal et al. 2018). High lipid peroxidation is a major feature of HD (Hatami et al. 2018). Low levels of GSH are also a feature of HD (Mason et al. 2013). In the 3-NP model of rats, it was found that the significant reduction of GSH was caused by downregulation of Nrf2 (Sayed et al. 2020).

Mitochondrial dysfunction, cellular antioxidants link Nrf2 to HD pathogenesis (Zgorzynska et al. 2021). Precisely through its regulatory role (e.g., GSH, GST), Nrf2 counteracts 3-NP-induced enhanced OS (Vasconcelos et al. 2019). Although the specific mechanism of Nrf2 during mtHtt formation and accumulation is in doubt (Zoungrana et al. 2022), neuroprotection and prolonged lifespan in animal models of HD through its activation of certain parts of the brain are clear (Tsvetkov et al. 2013; Zgorzynska et al. 2021). Nrf2-induced expression of p62 autophagy-associated protein and LC3 mediates rapid clearance of toxic mtHtt aggregates (Saito et al. 2020). Nrf2 reduces levels of pro-inflammatory cytokines and inflammatory mediators released by deposition of mtHtt aggregates through upregulation of HO1 (Dowling et al. 2010; Luo et al. 2018; Zgorzynska et al. 2021). The activity of NOX is also inhibited by Nrf2, which hinders ROS production (Benarroch 2017). Interestingly, Nrf2 can transcriptionally activate BDNF (Huang et al. 2020; Yao et al. 2021), while BDNF can influence Nrf2 translocation and subsequent activation to repair redox homeostasis (Bouvier et al. 2017). A study shows that naringin is neuroprotective against 3-NP-induced neurotoxicity in pheochromocytoma cells (PC12 cells) through PI3K-Akt pathway-mediated Nrf2 activation (Kulasekaran and Ganapasam 2015). In the nervous system, specific activation of Nrf2 in astrocytes can protect neurons (Calkins et al. 2010). Transplantation of Nrf2-overexpressing astrocytes and chemical activation of Nrf2 are protective against 3-NP- and malonate-induced striatal injury, respectively (Shih et al. 2005). In view of this, Nrf2 activation in astrocytes may be a viable treatment option for oxidative stress-related chronic neurodegeneration (Vargas and Johnson 2009).

## ALS

Amyotrophic lateral sclerosis (ALS), also known as Lou Gehrig's disease, is the third most common neurodegenerative disorder (Chiò et al. 2013) characterized by degeneration and loss of motor neurons in the cerebral cortex, brainstem, and spinal cord (Wijesekera and Nigel Leigh 2009). Among them, sporadic ALS (sALS) accounts for about 90%, and the possible causes are speculated to be: abnormal immune system, mitochondrial dysfunction, and exposure to toxic environmental chemicals (Martin et al. 2017); the incidence of familial ALS, which accounts for approximately 10%, is related to gene mutations, typically including: Cu^2+^/Zn^2+^ *superoxide dismutase 1* (*SOD1*) (Rosen 1993), *TAR DNA-binding protein 43* (*TDP-43* or *TARDBP*) (Sreedharan et al. 2008), *fused in Sarcoma* (*FUS*) (Vance et al. 2009), and *chromosome 9 open reading frame 72* (*C9orf72*) gene (DeJesus-Hernandez et al. 2011), whose function related to protein homeostasis and RNA metabolism. Mouse models, which express human mutant SOD1, TDP-43, and C9orf72, can mimic ALS-related progressive symptoms and motor neuron loss (Taylor et al. 2016). The degeneration of the upper motor neurons located in the cerebral cortex and the lower motor neurons distributed in the brainstem and spinal cord correspond to different clinical manifestations (George et al. 2022). As ALS progresses, mutated protein aggregates will be released by degenerating spinal motor neuron bundles and taken up into the cytoplasm by other neurons or glial cells (Taylor et al. 2016).

Studies have shown that the aggregation of a variety of abnormal proteins, also known as RNA-binding proteins (Blokhuis et al. 2013), including TDP or FUS, can lead to OS and neurotoxicity (Zuo et al. 2021). In neurons, the progression of ALS will affect RNA metabolism, mitochondrial function, cytoskeletal integrity, axonal transport dynamics, and cause increased neuroinflammation, glutamate excitotoxicity, ROS production (Bonafede and Mariotti 2017), while excessive OS eventually leads to neuronal meta death (Wood and Langford 2014). Gradual accumulation of cytotoxic misfolded protein aggregates and marked loss of motor neurons are similar features of the progression of sALS and fALS (Blokhuis et al. 2013). Aggregated TDP-43 blocks nucleoplasmic transport by disrupting the nuclear pore complexes (Chou et al. 2018). In ALS models, failure of oligodendrocyte precursors to differentiate and mature affects myelin formation in axons (Benarroch 2021). Given the high metabolic rate, active mitochondrial respiration, high activity of NOX, low levels of catalase and GSH, and reduced Nrf2 levels, motor neurons have increased sensitivity to OS (Baxter and Hardingham 2016; Liddell 2017; Brandes and Gray 2020; Izrael et al. 2020). Abnormal oxidative damage, such as signs of lipid peroxidation, was found in biological samples and autopsy tissues from patients with sALS or fALS (Blasco et al. 2017), and was similarly confirmed in ALS cells and animal models (Pedersen et al. 1998; Ferraiuolo et al. 2011; Johnson and Johnson 2015; Moujalled et al. 2017). In ALS mouse models and ALS patients, the GSSG/GSH ratio, an indicator of OS, was also increased (Vargas et al. 2011; Weiduschat et al. 2014; Blasco et al. 2017). Relatedly, in mutant SOD1 mice, GSH synthesis-associated System Xc^−^ dysregulation may cause GSH depletion (Mesci et al. 2015). In particular, aggregation of SOD1 associated with some fALS was shown to be promoted by lipid peroxidation (Dantas et al. 2020). Evidence of iron accumulation was found both in the spinal cord and brain regions of ALS patients (Kasarskis et al. 1995; Moreau et al. 2018) and in neurons of ALS mouse models (Lee et al. 2015; Moreau et al. 2018). In addition, iron-binding protein abnormalities were found in the ALS mouse models (Wang et al. 2022d). Markers of ferroptosis in the blood, such as ferritin, Tf and lipid peroxides, are also associated with ALS prognosis (Devos et al. 2019). Vice versa, CuATSM, a potent inhibitor of ferroptosis (Southon et al. 2020), has a robust and reliable rescue effect in ALS mouse models (Soon et al. 2011; Roberts et al. 2014). Positive effects of two iron chelators, desferrioxamine and deferiprone, were reported for SOD1^G93A^ mice and for SOD1^G86R^ mice and ALS patients, respectively (Lee et al. 2015; Moreau et al. 2018). Substantial evidence links iron accumulation (Oba et al. 1993; Kasarskis et al. 1995; Jeong et al. 2009), imbalances in the antioxidant system (Chi et al. 2007; Choi et al. 2015), and lipid peroxidation (Ferrante et al. 1997; Pedersen et al. 1998), among others, to ferroptosis in ALS-affected CNS regions. Correspondingly, Nrf2, an important regulator of ferroptosis, was found to be attenuated in ALS (Kirby et al. 2005; Wood-Allum et al. 2006), and its reduced activity was shown to be a hallmark of ALS in human biosamples (von Otter et al. 2010; Bergström et al. 2014). Similarly, some studies have also found that in skeletal muscle of an ALS mouse model, increased Nrf2 levels occur at very early time points, even before the onset of movement impairments (Kraft et al. 2007), which may provide support for the hypothesis of motor neuron degeneration starting at the level of the neuromuscular junction (Pavlovskiĭ and Mikaĭlenko 1976). Whereas, lower activation levels of Nrf2 occur in spinal motor neurons and astrocytes during symptoms (Kraft et al. 2007). In NSC-34 SOD1^G93A^ cells, overexpression of Nrf2 can significantly improve OS and reduce cell death (Nanou et al. 2013). Likewise, in SOD1^G93A^ mice, Nrf2 levels were reduced in primary motor neuron cultures compared to wild-type mice in the same litter (Pehar et al. 2007). Interestingly, ALS progression was not significantly affected by Nrf2 loss in multiple SOD1 mouse models (SOD1^G93A^, SOD1^G85R^ or SOD1^H46R^) (Vargas et al. 2013; Guo et al. 2013b; Hadano et al. 2016). Further, in the motor neurons of SOD1^G93A^ mice, overexpression of Nrf2 only altered disease onset but not the survival (Vargas et al. 2013). This could be partly explained by the limited protection of Nrf2 against disease markers in backcross experiments between ALS mice and Nrf2-null mice, presumably as a result of compensatory mechanisms (Vargas et al. 2013; Guo et al. 2013b). In addition, studies on lymphoblastoid cells from ALS patients suggest that Nrf2-mediated antioxidant responses may only be present in a specific ALS, namely sALS, but this conclusion remains to be further studied (Lastres-Becker et al. 2021). Mutations in p62, also known as autophagy carrier protein SQSTM1, which can form a positive feedback loop with Nrf2, are associated with ALS and frontotemporal dementia, as previously described (Foster et al. 2020). Among them, the STGE motif contained in p62 can exhibit a high affinity for Keap1 after phosphorylation by signaling kinases such as casein kinase 1 (CK1), transforming growth factor β-activated kinase 1 (TAK1), mTORC1 and PKCσ similar to the ETGE motif of Nrf2 (Ichimura et al. 2013; Hashimoto et al. 2016; Watanabe et al. 2017; Jiang et al. 2017). Among novel genes associated with ALS, 10 *SQSTM1* gene (encoding p62) mutations were found in both sporadic and familial forms of ALS, and 8 of 9 missense variants were classified as pathogenic by predictive in silico analysis (Jiménez-Villegas et al. 2021). Interestingly, in the SOD1^H46R^ mouse model, overexpression of p62 instead accelerated the onset of ALS and resulted in shortened lifespan (Mitsui et al. 2018). Due to sporadic and SOD1-associated motor neuron degeneration in the spinal cord, Nrf2-regulated doredoxin 3 (Prx3) is downregulated, suggesting that impaired mitochondrial antioxidant defense is a replacement mechanism for intracellular OS (Wood-Allum et al. 2006). Urate (2,6,8-trioxy-purine), proposed as a prognostic factor for survival in ALS patients due to its reduced levels in the serum of ALS patients (Keizman et al. 2009), is also an important endogenous antioxidant in the body (Sautin and Johnson 2008) that activates Nrf2 by inhibiting GSK3β through Akt (Zhang et al. 2019a). As the role of non-cell-autonomous pathogenic mechanisms has come to the fore, glial cells, dominated by stellate glial cells, play a more prominent role in ALS pathology (Birger et al. 2019; Izrael et al. 2020; Van Harten et al. 2021). Nrf2 in astrocytes activates non-cell-autonomous protection of nearby neurons and improves OS by mediating antioxidant responses (Yamanaka et al. 2008; Vargas et al. 2008; Haidet-Phillips et al. 2011; Liddell 2017; Kim 2021). GSH produced by activated Nrf2 pathway in the brain is mainly derived from astrocytes (Vargas et al. 2006), whose neuroprotective function depends on the transport of GSH precursors from astrocytes to motor neurons (Andronesi et al. 2020; Aoyama 2021) (Fig. 3d). Similarly, activated Keap1–Nrf2–ARE pathways induce the expression of target genes in glial cells at a late stage but not in spinal motor neurons (Mimoto et al. 2012). In the SOD1^G93A^ rat model, Nrf2 levels of astrocytes at the onset of ALS were found to be higher than basal levels in controls (Vargas et al. 2005; Kraft et al. 2007). In contrast, under some circumstances, astrocytes may become reactive and contribute to the neuroinflammatory response, which in turn promotes neurodegeneration (Aoyama 2021). This reactivity also occurs after brain injury (Tripathi et al. 2017), which is considered to be a trigger or a link to ALS (Anderson et al. 2018). The role of astrocytes reactivity in the pathogenesis of ALS has been demonstrated by in vitro cell co-culture (Haidet-Phillips et al. 2011; Ferraiuolo et al. 2011; Tripathi et al. 2017). Nevertheless, also in in vitro co-culture experiments (Vargas et al. 2005), overexpression or chemical activation of Nrf2 in astrocytes was found to reduce neuronal death, and consistent findings were found in ALS mouse models (Vargas et al. 2008). Other glial cells, such as microglia, can also be involved in motor neuron degeneration (Brites and Vaz 2014). Given the higher than neuronal levels of Nrf2 in astrocytes and microglia in the brain (Boas et al. 2021), activation of Nrf2-associated pathways may be a potential treatment options to suppress microglia-mediated inflammatory responses (Li et al. 2018; Saha et al. 2022).

## Biomarkers and therapeutics targeting Nrf2 and ferroptosis in NDs

In NDs, increasing evidence has been found for ferroptosis, which has been used to explain the pathogenesis of NDs. During this process, detection and monitoring of ferroptotic responses inevitably became one of the research priorities. Indicators of iron homeostasis include intracellular or mitochondrial Fe^2+^ levels, NCOA4 (Hou et al. 2016) that mediates ferritin degradation, TFRC in cell cultures or tissues, and anti-TFRC antibodies (known as 3F3-FMA) (Feng et al. 2020) that suggest ferroptosis or related damage. Significant markers involved in lipid peroxidation include NOX, arachidonic acid lipoxygenase (ALOX) isoforms. Importantly, upregulated ACSL4 is a sensitive marker reflecting ferroptosis (Yuan et al. 2016). In addition, there are lipid peroxides (PUFA-OOH, PL-OOH) (Kagan et al. 2017) and end products [MDA (Ye et al. 2020), 4-HNE (Shintoku et al. 2017) etc.], markers of oxidative DNA damage Compound (8OH-dG) can detect and quantify the degree of lipid peroxidation in ferroptosis (Zhang et al. 2019c; Dai et al. 2020a). Some researches indicate that these biomarkers can be detected in relevant experimental models and patient tissues (Table 2).

**Table 2:** Experimental models and patients with neurodegenerative diseases in studies related to ferroptosis

Disease	Models and patients	Outcome	References
PD	FAC-induced PC12-NGF cell	GPX4, FTH1 ↓ DMT1, TfR1, FPN, ACSL4, ROS↑	Zeng et al. (2021)
PD	6-OHDA-induced PC12 cell	GPX4, FTH1 ↓ NCOA4, α-Syn ↑	Tian et al. (2020)
PD	Rat model of Rotenone-induced SNpc injury	GSH, SOD, GPX4 ↓ MDA↑	Avci et al. (2021)
AD	Senescence-accelerated P8 (SAMP8) mice with cognitive impairment	SOD, GSH, FTH1, Nrf2, GPX4↓ Fe^2+^, MDA, TfR1, NCOA4, ROS ↑	Shao et al. (2021)
PD	Primary dopaminergic neurons of mice induced by 1-methyl-4-phenylpyridinium (MPP^+^)	GPX4, GSH ↓ Nrf2, ROS↑	Wang et al. (2022c)
AD	Male APPswe/PSEN1dE9 (APP/PS1) double transgenic AD mice	FTH, GPX4, Nrf2, NOQ1 ↓ DMT1, ALOX5, TFRC ↑	Wang et al. (2022a)
AD	APP/PS1 mice	GSH, GPX4 ↓ ROS, DMT1, ACSL4, NCOA4 ↑	Gao et al. (2021)
ALS	SOD1^G93A^ mouse model	MDA ↑	Wang et al. (2022e)
ALS	Post-mortem brain and spinal cord tissues of ALS patients and SOD1^G93A^ mouse model	8-Dihydroguanosine (8-OHG) ↑	Chang et al. (2008)
ALS	Post-mortem specimens of lumbar spinal cord and/or occipital cortex	4-HNE ↑	Pedersen et al. (1998)
ALS	Motor cortex of ALS patients	GSH ↓	Weiduschat et al. (2014)
HD	NLS-N171-82Q transgenic mouse model	Nrf2 (striatum) ↓	Chaturvedi et al. (2010)
HD	Doxycycline-induced rat PC12 cell lines	GCH1 ↓	Van Roon-Mom et al. (2008)

*Nfe2l2* is an iron death-associated gene, and although its pathway activation receives influence from other signaling molecules (e.g., Keap1), its over-regulated target gene reflects to some extent the increased OS damage of ferroptosis. These target genes are involved in iron metabolism, GSH metabolism, antioxidant response, etc. However, it is difficult to determine the specific effect of Nrf2 on ferroptosis based on the expression of Nrf2 target genes alone (Chen et al. 2021). For example, GSH, CoQ_10_ and BH_4_, which Nrf2 depends on for its antioxidant effect, are not conducive to the study of specific molecular mechanisms of ferroptosis due to their broad-spectrum effects (Chen et al. 2021). Nrf2-associated markers of ferroptosis require binding to others for specific detection and detection of cellular ferroptosis. Nevertheless, according to several studies (Habas et al. 2013), Nrf2 has become an important therapeutic target of NDs because of its important role in protecting neurons from OS and glutamate-induced excitotoxicity and ultimately maintaining the survival of injured neurons (Zhang et al. 2023).

As previously mentioned, brain iron deposition has been found in a variety of ND. Studies have shown that iron chelators such as DFO, deferiprone (DFP) and cyclopiroxamine (CPX) further prevent iron-dependent OS and ultimately inhibit iron death by binding to ferrous iron to reduce intracellular iron overload (Taher et al. 2017). Clinically, DFP, as an oral hydroxypyridone derivative, is used in the treatment of diseases such as Friedreich's ataxia and thalassemia (Aminzadeh-Gohari et al. 2020). The pathogenesis of several NDs also involves mitochondrial damage, and prevention of lipid peroxidation by targeted scavenging of ROS produced in mitochondria has become a new therapeutic idea (Trnka et al. 2008). Such mitochondria-targeted antioxidants include Mitoquinone (MitoQ), MitoVitE (vitamin E), MitoPBN (CoQ and phenyl tert-butyl nitrone conjugate), and MitoTEMPO (SOD mimetic). When referring to antioxidant effects, it is equally appropriate to address natural and synthetic antioxidants, such as: retinol (vitamin A), ascorbic acid (vitamin C), α-tocopherol (vitamin E) and its water-soluble derivatives trolox, N-acetylcysteine (NAC), uric acid, some of the target molecules of Nrf2 (GSH, CoQ). However, the activity of these antioxidants depends on the metabolic or redox state of the cell/organism, exerting antioxidant or pro-oxidant effects (Angelova and Müller 2006, 2009) (Table 3).

**Table 3:** Preclinical and clinical studies of promising ferroptosis

Compound	Mechanism	Disease and damage	Model and trial	Outcome	References
DFO	Iron chelator (reduces intracellular free ferrous ion levels → inhibits iron overload)	PD	Ammonium ferric citrate (FAC)-treated	GPX4, FTH1↑	Zeng et al. (2021)
			PC-NGF cells	DMT1, TfR1, FPN, ACSL4, ROS↓	
DFP		General anesthesia (GA)	Ketamine- and sevoflurane treated; hippocampal neurons	GSH, SOD↑	Jing et al. (2020)
				ROS, MDA, DMT1↓	
CPX		Ferroptosis	Glutamate-treated rat organotypic hippocampal slice culture (OHSC)	ROS↓	Dixon et al. (2012)
MitoQ	Radical Trapping Antioxidant (RTA, Targets mitochondrial ROS scavenging → rescues mitochondrial integrity and function)	Oxidative cell death	RSL3-treated neuronal HT22 cells	Mitochondrial ROS, lipid peroxidation↓	Jelinek et al. (2018)
VitE	ALOX inhibitor (inhibits 15-LOX expression, iron accumulation, and lipid peroxidation)	Epilepsy	Pentylenetetrazol (PTZ)-treated chronic epileptic rat model	In the hippocampus	Zhang et al. (2022b)
				GSH, GPX4↑	
				Iron accumulation, 15-LOX, ROS, MDA↓	
NAC	ALOX inhibitor (inhibits toxic arachidonic acid products of nuclear ALOX5)	Intracerebral hemorrhage (ICH)	Collagenase-treated mice; Hemin-treated primary cortical neurons	GSH↑	Karuppagounder et al. (2018)
				ALOX5↓	
Curcumin	Activates Nrf2–HO1 pathway; rescues ethanol induced activated astrocytes and microglia, in vivo and in vitro; may inhibit ethanol-induced apoptotic cell death	Ethanol-induced neurodegeneration and memory impairment	Ethanol-treated male mice	Nrf2, HO1↑	Ikram et al. (2019)
				Lipid peroxidation, ROS, p-JNK, IL-1β, COX-2, Caspase-3, Bax, PARP-1, TLR4/RAGE and its downstream inflammatory mediators (TNF-α, p-Nf-κB), Markers of astrocyte and microglial activation (GFAP, Iba-1)↓	
	Nrf2–HO1 pathway (activates Nrf2–HO1 pathway → inhibit the ROS production)	ICH	Collagenase-treated mice; erastin-treated HT22 murine hippocampal cells	GSH, Nrf2, HO1↑	Yang et al. (2021)
				ROS↓	
Resveratrol	RTA	Oxygen–glucose deprivation/reoxygenation (OGD/R) and ischemic injury	Middle cerebral artery occlusion/reperfusion (MCAO/R)-treated adult male SD rat; OGD/R-treated primary cortical neuron	GSH, GPX4, ferritin↑	Zhu et al. (2022)
				ACSL4, ROS↓	
Sulforaphane	Nrf2 activator	ICH	Nrf2^−/−^ and WT mice and rats subjected to autologous blood injection; microglia	SOD1, GST, catalase, NQO1, HO1↑	Zhao et al. (2015)
DMF	Activates the Nrf2–SLC7A11–HO1 axis	Hepatic ischemia–reperfusion injury	Liver IRI mouse model hypoxia–reoxygenation injury (HRI) alpha mouse liver (AML12) cells	GSH/GSSG, GPX4, Nrf2, SLC7A11, HO1↑	Qi et al. (2023)
				MDA, COX2↓	
4-Octyl itaconate	Activates Keap1–Nrf2 signaling via Alkylated cysteine residues of Keap1	H_2_O_2_-induced oxidative damage	OI-treated SH-SY5Y neuronal cells and epigenetically de-repressed (by TSA treatment) primary murine neurons	HO1, NQO1, GCLC, Ninj2↑	Liu et al. (2018)
				Cleavages of PARP, caspase-3 and caspase-9, ROS, superoxide accumulation ↑	
Salidroside	Activates Nrf2–HO1 pathway	AD	Aβ1 − 42-induced AD mice and glutamate-injured HT22 cells	In vitro: GPX4, SLC7A11, GSH, SOD, Nrf2↑	Yang et al. (2022a)
				LPO, ROS, MDA↓	
				In vivo: GPX4, HO1, NQO1↑	

Numerous studies have reported that bioactive phytochemicals can modulate ferroptosis, with flavonoids and polyphenols antagonizing ferroptosis by participating in iron metabolism and the Nrf2 pathway (Zheng et al. 2021) (Table 3). Curcumin, a natural polyphenol, is also a effective inhibitor of ferroptosis (Kajarabille and Latunde-Dada 2019), can directly inhibit lipid peroxidation (Sreejayan and Rao 1994), and has neuroprotective effects, showing promising prospects in the prevention or treatment of neurological diseases such as HD and PD (Hong et al. 2004). Resveratrol, a stilbene polyphenol derived from grape (*Vitis vinifera*) skin, inhibits NF-κB (Reinisalo et al. 2015) and directly inhibits lipid peroxidation by scavenging free radicals (Tadolini and Franconi 1998).The previously mentioned sulforaphane, curcumin and resveratrol can activate Nrf2 by covalently modifying Keap1 (Cores et al. 2020), which has advantages over synthetic compounds due to its minimal side effects of antioxidant and anti-inflammatory effects. Therefore, it is widely used in the treatment of many diseases (Gugliandolo et al. 2020).

In addition to electrophilic inhibitors, Keap1 inhibitors include protein–protein interaction (PPI) inhibitors (Bono et al. 2021), which competitively inhibit the protein–protein interaction between Keap1 and Nrf2 through non-electrophilic non-covalent compounds, developed to address off-target toxicity from electrophilic compounds (Robledinos-Antón et al. 2019). A typical PPI is the clinically approved Nrf2 activator, dimethyl fumarate (DMF), for the treatment of relapsing–remitting multiple sclerosis (Blair 2019), which at nanomolar concentrations modifies specific reactive cysteine (Unni et al. 2021). Studies have shown that DMF has a protective effect on neurons from neurotoxin-induced oxidative damage and is used in animal models of AD (Ahuja et al. 2016; Lastres-Becker et al. 2016). Similarly, another novel drug that activates Nrf2 by alkylating Keap1 is 4-octyl itaconate, by which it protects primary neurons against H_2_O_2_-induced cell death (Liu et al. 2018). In addition, there are other ways to activate the Nrf2 pathway. *BACH1*, which suppresses the transcriptional regulation of Nrf2 by competitively binding to AREs, is also a promising therapeutic target (Hushpulian et al. 2021). p62, which promotes the degradation of Keap1 (Mizunoe et al. 2018), and GSK3β, which participates in the degradation of Nrf2, may be potential therapeutic targets. As mentioned previously, activation of GSK3β has been mentioned in various NDs and several GSK3β inhibitors have entered testing in clinical trials related to AD and ALS (Choi et al. 2020). Of note, the FDA-approved non-phenolic antioxidant edaravone has been preclinically shown to increase Nrf2 levels in ALS patients (Ohta et al. 2019; Zhang et al. 2019b). In a chronic social defeat stress (CSDS) depression model mouse, edaravone mediates the silent message regulator 2 homolog 1 (Sirt1)–Nrf2–HO1–GPX4 pathway to inhibit hippocampal neuronal ferroptosis (Dang et al. 2022) (Table 3).

## Outlook and prospects

Accumulating evidence links ferroptosis to ND pathology. As the disease progresses, damage to the antioxidant system, excessive OS, and altered Nrf2 expression levels, especially the inhibition of ferroptosis by lipid peroxidation inhibitors and adaptive enhancement of Nrf2 signaling, suggest that Nrf2 is essential for the detection and identification of ferroptosis and potential clinical implications of targeted therapy for neuronal loss and dysfunction.

To better understand the correlation of ferroptosis with neurodegenerative disease, the occurrence requires the combination with other biomarkers. The lack of unique biomarkers for ferroptosis hinders its assessment in ND, limiting in-depth studies of its mechanisms in neurodegeneration and therapeutic approaches targeting ferroptosis. Therefore, finding specific biomarkers of ferroptosis is still an urgent problem to be solved.

Nrf2 is widely recognized as a major therapeutic target in ND. Some natural bioactive components act as Nrf2 activators to regulate ferroptosis. Different from classical ferroptosis regulators, they have the advantages of stable structure, higher safety and less cost. Electrophilic inhibitors of Keap1 have side effects due to off-target effects, and the resulting PPI inhibitors have been extensively studied and have broad clinical potential. Other Nrf2 activators, such as GSK3β inhibitors, may have adverse side effects by affecting other signaling and metabolic pathways. Some of these Nrf2 activators are in clinical trials, such as curcumin and resveratrol.

In addition to the requirements of safety, specificity, stability and high bioavailability, high BBB permeability is a necessary condition for Nrf2 activators to enter clinical application. For this reason, DMF remains, as of now, the only drug approved by the FDA for the neurodegenerative disease (relapsing–remitting multiple sclerosis). Conjugating relevant drugs to BBB-penetrating peptides or loading them into nanoparticles or liposomes are possible development directions (Bertrand et al. 2011; Furtado et al. 2018).

The transformation of basic research results is the current challenge. It should be pointed out that the expression of Nrf2 target genes differs between neurons and glial cells (especially astrocytes), with glial cells predominating. However, the specific mechanism of the lipid peroxide-induced cell death cascade downstream of ferroptosis is still unclear, and the regulation of Nrf2-related pathways is still one of the best ways to treat ND. In summary, we focus on iron death in a neurological context, the key regulatory role of Nrf2 on iron death, and discuss that the close link between Nrf2 and ferroptosis is of clear and important significance for understanding the mechanism of ND based on OS, characterized by neuronal loss and mitochondrial dysfunction, and represented by AD, PD, HD, and ALS, and point out the bright prospect of Nrf2-targeted therapy in the diagnosis and treatment of ND.
